# Disentangling the mechanisms shaping the surface ocean microbiota

**DOI:** 10.1186/s40168-020-00827-8

**Published:** 2020-04-20

**Authors:** Ramiro Logares, Ina M. Deutschmann, Pedro C. Junger, Caterina R. Giner, Anders K. Krabberød, Thomas S. B. Schmidt, Laura Rubinat-Ripoll, Mireia Mestre, Guillem Salazar, Clara Ruiz-González, Marta Sebastián, Colomban de Vargas, Silvia G. Acinas, Carlos M. Duarte, Josep M. Gasol, Ramon Massana

**Affiliations:** 1 Institute of Marine Sciences (ICM), CSIC, 08003 Barcelona, Catalonia Spain; 2 Department of Biosciences, Section for Genetics and Evolutionary Biology, University of Oslo, 0316 Oslo, Norway; 3 Laboratory of Microbial Processes & Biodiversity (LMPB), Department of Hydrobiology (DHB), Universidade Federal de São Carlos (UFSCar), São Carlos, 13565-905 SP Brazil; 4 Institute for the Oceans and Fisheries, University of British Columbia, 2202 Main Mall, Vancouver, BC V6T 1Z4 Canada; 5 European Molecular Biology Laboratory, Meyerhofstr. 1, 69117 Heidelberg, Germany; 6 Sorbonne Universités, UPMC Univ Paris 06, CNRS UMR 7144, Adaptation et Diversité en Milieu Marin, Equipe EPEP, Station Biologique de Roscoff, 29680 Roscoff, France; 7 Centro de Investigación Oceanográfica COPAS Sur-Austral, Departamento de Oceanografía, Universidad de Concepción, Concepción, Chile; 8 Centro FONDAP de Investigación Dinámica de Ecosistemas Marinos de Altas Latitudes (IDEAL), Universidad Austral de Chile, Valdivia, Chile; 9 Department of Biology, Institute of Microbiology and Swiss Institute of Bioinformatics, ETH Zürich, 8093 Zürich, Switzerland; 10 Oceanography and Global Change Institute, IOCAG, University of Las Palmas de Gran Canaria, ULPGC, 35214 Gran Canaria, Spain; 11 King Abdullah University of Science and Technology (KAUST), Red Sea Research Center (RSRC), Thuwal, Saudi Arabia; 12 Centre for Marine Ecosystems Research, School of Science, Edith Cowan University, Joondalup, WA Australia

**Keywords:** Ocean, Plankton, Microbiota, Picoeukaryotes, Prokaryotes, Community structure, Ecological processes, Selection, Dispersal, Drift

## Abstract

**Background:**

The ocean microbiota modulates global biogeochemical cycles and changes in its configuration may have large-scale consequences. Yet, the underlying ecological mechanisms structuring it are unclear. Here, we investigate how fundamental ecological mechanisms (*selection*, *dispersal* and *ecological drift*) shape the smallest members of the tropical and subtropical surface-ocean microbiota: prokaryotes and minute eukaryotes (picoeukaryotes). Furthermore, we investigate the agents exerting abiotic selection on this assemblage as well as the spatial patterns emerging from the action of ecological mechanisms. To explore this, we analysed the composition of surface-ocean prokaryotic and picoeukaryotic communities using DNA-sequence data (16S- and 18S-rRNA genes) collected during the circumglobal expeditions *Malaspina*-*2010* and *TARA*-*Oceans*.

**Results:**

We found that the two main components of the tropical and subtropical surface-ocean microbiota, prokaryotes and picoeukaryotes, appear to be structured by different ecological mechanisms. Picoeukaryotic communities were predominantly structured by dispersal-limitation, while prokaryotic counterparts appeared to be shaped by the combined action of dispersal-limitation, selection and drift. Temperature-driven selection appeared as a major factor, out of a few selected factors, influencing species co-occurrence networks in prokaryotes but not in picoeukaryotes, indicating that association patterns may contribute to understand ocean microbiota structure and response to selection. Other measured abiotic variables seemed to have limited selective effects on community structure in the tropical and subtropical ocean. Picoeukaryotes displayed a higher spatial differentiation between communities and a higher distance decay when compared to prokaryotes, consistent with a scenario of higher dispersal limitation in the former after considering environmental heterogeneity. Lastly, random dynamics or *drift* seemed to have a more important role in structuring prokaryotic communities than picoeukaryotic counterparts.

**Conclusions:**

The differential action of ecological mechanisms seems to cause contrasting biogeography, in the tropical and subtropical ocean, among the smallest surface plankton, prokaryotes and picoeukaryotes. This suggests that the idiosyncrasy of the main constituents of the ocean microbiota should be considered in order to understand its current and future configuration, which is especially relevant in a context of global change, where the reaction of surface ocean plankton to temperature increase is still unclear.

Video Abstract

## Background

The surface ocean microbiota is a pivotal underpinning of global biogeochemical cycles [1, 2]. The smallest ocean microbes, the picoplankton, have a key role in the global carbon cycle, being responsible for an important fraction of the total atmospheric carbon and nitrogen fixation in the ocean [3–5], which supports ≈ 46% of the global primary productivity [6]. Oceanic picoplankton plays a fundamental role in processing organic matter by recycling nutrients and carbon to support additional production as well as by channelling organic carbon to upper trophic levels through food webs [5, 7, 8]. The ocean picoplankton includes prokaryotes (both bacteria and archaea) and tiny unicellular eukaryotes (hereafter picoeukaryotes), which feature fundamental differences in terms of cellular structure, feeding habits, metabolic diversity, growth rates and behaviour [9]. Even though marine picoeukaryotes and prokaryotes are usually investigated separately, they are intimately connected through biogeochemical and food web networks [10–12].

The underlying ecological mechanisms determining the biogeography of prokaryotes and picoeukaryotes in the global ocean are unclear [13, 14]. In particular, we do not know whether these crucial components of the ocean microbiota are structured by the action of the same or different ecological processes. Comprehending such processes is fundamental, as their differential action can produce changes in the ocean microbiota composition that could impact global ecosystem function [15–17]. A recent ecological synthesis explains the structure of communities and the emergence of biogeography as a consequence of the action of four main processes: *selection*, *dispersal*, *ecological drift* and *speciation* [18]. Selection involves deterministic reproductive differences among individuals from different or the same species as a response to biotic or abiotic conditions. Selection can act in two opposite directions; it can constrain (*homogeneous selection*) or promote (*heterogeneous selection*) the divergence of communities [19]. Dispersal is the movement of organisms across space, and rates can be high (*homogenising dispersal*), moderate, or low (*dispersal limitation*) [19]. Dispersal limitation occurs when species are absent from suitable habitats because potential colonizers are too far away [20], and the significance of dispersal limitation increases as geographic scale increases [21]. Ecological drift (hereafter *drift*) in a local community refers to random changes in species’ relative abundances derived from stochastic birth, death, offspring production, immigration and emigration [18]. The action of drift in a *metacommunity*, that is, local communities that are connected via dispersal of multiple species [22], may lead to neutral dynamics [21], where random dispersal is the main mechanism of community assembly. Finally, speciation is the evolution of new species [18], and it will not be considered hereafter as it is expected to have a small impact in the turnover of communities that are connected via dispersal [23], being also difficult to measure this ecological process in the wild.

The action of the previous ecological processes is typically manifested as different taxonomic or phylogenetic patterns of community turnover, that is, β-diversity. At the moment, there are several estimators of β-diversity which capture different aspects of community turnover [24]. Most of these indices consider taxonomic or phylogenetic aspects of communities, but not species-association patterns, which can also manifest the action of ecological processes. For example, selection exerted by an environmental variable can drive species co-occurrences generating groups of highly associated species or modules in association networks that correspond with specific environmental conditions [25]. Different members of these modules may be more abundant in specific regions of the ocean, contributing to increase β-diversity estimates between these regions when based on standard compositional or phylogenetic β-diversity metrics. Yet, β-diversity estimates based on association-aware metrics may point to higher similarity between these regions, as taxa belong to the same modules. Furthermore, modules may display correlations with environmental heterogeneity. Thus, association aware metrics of β-diversity may allow unveiling community patterns and their relationships with environmental variables (i.e. selection), which would be missed by standard approaches [26]. So far, most studies investigating the structure of the ocean microbiota have not considered species associations in their analyses of β-diversity.

The differential action of selection, dispersal and drift may generate different microbial assemblages that could feature diverse metabolisms and ecologies [16, 17]. Moderate or high selection together with moderate dispersal rates may couple environmental heterogeneity with combinations of species, leading to a spatial pattern known as *species sorting* [27]. In contrast, high or low levels of dispersal may decouple environmental heterogeneity (i.e. selection) from the composition of species assemblages. High dispersal rates may maintain populations in habitats to which they are maladapted [16, 22]. Inversely, low dispersal rates may promote microbial assemblages that become more different as the geographic distance between them increases (*distance decay*). If environmental heterogeneity and geographic distance covary, then distance decay could reflect both selection and dispersal limitation [28]. Drift is expected to cause important random effects in local community composition in cases where selection is weak and populations are small [15, 29].

Here, we investigate the mechanisms that shape the smallest members of the surface-ocean microbiota by using DNA-sequence data collected in two of the largest circumglobal oceanographic expeditions to date, *Malaspina 2010* [30] and *TARA Oceans* [31]. Specifically, we ask: What is the relative importance of selection, dispersal and drift in structuring the sunlit ocean microbiota? Do these processes act similarly on main components of this microbiota (prokaryotes and picoeukaryotes)? What are the main agents that exert abiotic selection? Do species association networks reflect the action of selection in the upper ocean microbiota? What are the main spatial-structure patterns that emerge due to the action of selection, dispersal and drift?

## Results

### Quantifying the mechanisms that structure the surface ocean picoplankton

**Fig. 1:**
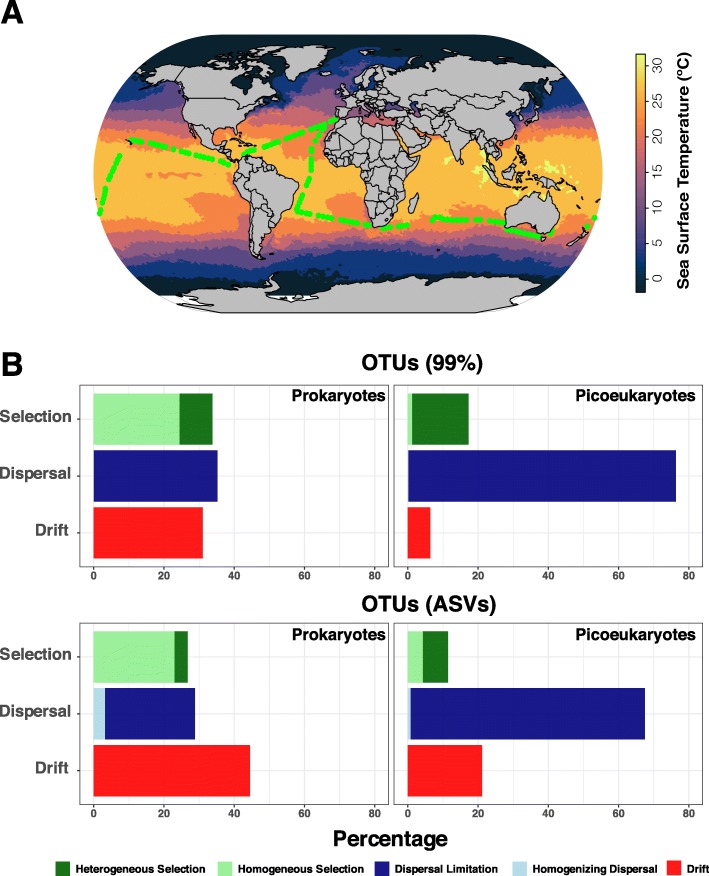
Ecological mechanisms shaping the tropical and subtropical surface-ocean picoplankton. **a** Position of the 120 stations included in this work that were sampled as part of the *Malaspina*-*2010* expedition (green dots) in the tropical and subtropical ocean. A snapshot of the global sea surface temperature, a main environmental driver affecting microbial distributions, is shown as a general representation of the temperature gradients in the surface ocean (as inferred using the ‘optimum interpolation sea surface temperature’ dataset from the NOAA corresponding to the 17 of March of 2018). Note that temperatures measured in situ were used in all analyses, not the ones displayed here. **b** Percentage of the community turnover associated to different ecological processes in prokaryotes and picoeukaryotes in the tropical and subtropical upper ocean as calculated using OTUs_-99%_ and OTUs_-ASVs_. Note that percentage refers to the percentage of pairs of communities that appear to be driven by a given process

We analysed 16S and 18S rRNA-genes from prokaryotes and picoeukaryotes in 120 globally distributed tropical and subtropical stations sampled during the *Malaspina 2010* expedition [30] (Fig. 1a; Figure S1, Additional file 1). *TARA Oceans* data were not included in these analyses as the type of generated DNA fragments could not be used for phylogenetic reconstructions (see details in ‘Methods’ section). Operational taxonomic units were delineated at 99% similarity (OTUs_-99%_) and as unique sequence variants (OTUs_-ASVs_, the maximum resolution for the 18S and 16S rRNA-gene). Analyses using both, OTUs_-99%_ and OTUs_-ASVs_ indicated that dispersal limitation was the dominant factor structuring picoeukaryotic communities, explaining ≈ 76–67% of community turnover, while this process had a lower importance in prokaryotes **(**≈ 35–25%; Fig. 1b). Note that percentage refers to the percentage of pairs of communities that appear to be driven by dispersal limitation. In contrast, homogenising dispersal had a very limited role in the structuring of the tropical and subtropical upper-ocean microbiota (< 3% for both picoeukaryotes and prokaryotes). Drift had a limited role in the structuring of picoeukaryotic communities as indicated by both OTUs_-99%_ and OTUs_-ASVs_, representing ≈ 21–6% of community turnover (Fig. 1b). In contrast, drift appeared as a relevant factor structuring prokaryotic communities, explaining ≈ 44–31% of the community turnover according to OTUs_-99%_ and OTUs_-ASVs_ (Fig. 1b). The role of selection was higher in prokaryotes compared to picoeukaryotes according to both OTUs_-99%_ and OTUs_-ASVs_, explaining ≈ 34–27% of the turnover of prokaryotic communities, and ≈ 17–11% of that in picoeukaryotes (Fig. 1b). Heterogeneous selection had a relatively higher importance in structuring picoeukaryotes as compared to prokaryotes (≈ 16–7% vs. ≈ 9–4%, respectively). Instead, homogeneous selection appeared more important in structuring prokaryotic (≈ 24–23%) than picoeukaryotic (≈ 1–4%) communities (Fig. 1b).

Our quantifications indicated different roles of ecological processes in structuring communities of marine prokaryotes and picoeukaryotes populating the tropical and subtropical surface-ocean (Fig. 1b). We then aimed at confirming these results using other more traditional approaches. In these analyses, considering *Malaspina* data, we used OTUs_-99%_, given that these likely correspond to well-defined lineages, while OTUs_-ASVs_ may reflect, in some cases, intraspecific variation [32]. We found moderate correlations between picoeukaryotic and prokaryotic β-diversity (Bray-Curtis: *ρ* = 0.58, gUniFrac: *ρ* = 0.61, *p* = 0.01, Mantel tests; Figure S2, Additional file 2). Given that rare species tend to occupy less sites than more abundant ones [33], communities featuring different proportions of abundant or rare species may display different spatial turnover. We found that picoeukaryotes had proportionally more regionally rare (i.e. mean abundances across all samples < 0.001%) species than prokaryotes (71% vs. 48% respectively) (Table S1, Additional file 3). This is consistent with the observation that picoeukaryotes had more restricted species distributions (i.e. occurring in < 20% of the stations) than prokaryotes (95% vs. 88% of the species respectively) (Figure S3, Additional file 4, Table S2, Additional file 5).

### Selection acting on the microbiota

**Fig. 2:**
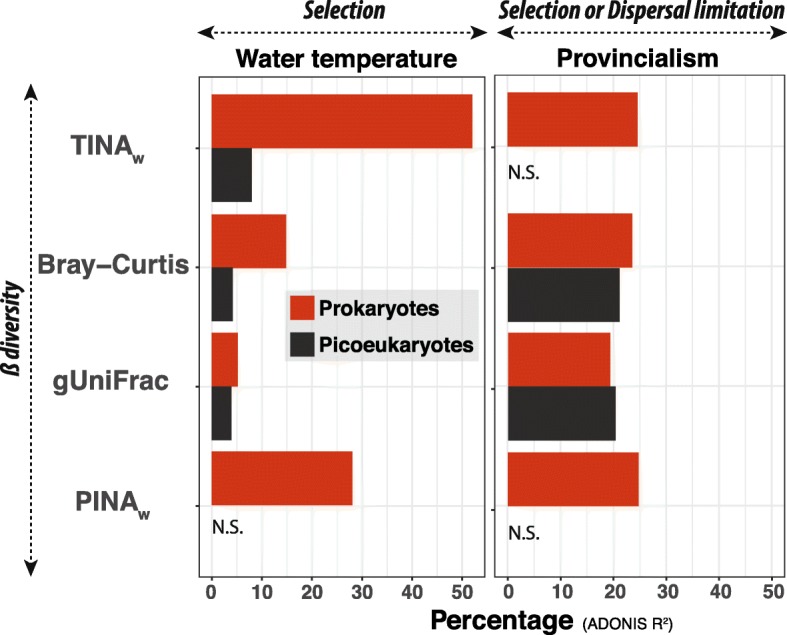
Main variables influencing the structure of the surface-ocean microbiota as captured by different β-diversity metrics. Percentage of variance in picoeukaryotic and prokaryotic community composition (ADONIS *R*^2^) explained by water temperature and Longhurst Provinces when using different β-diversity metrics. Figure based on the *Malaspina Meta*-*119* dataset (see ‘Methods’ section). *TINA*_*w*_ TINA weighted, *gUniFrac* generalized Unifrac, *PINAw* PINA weighted, *N*.*S*. non-significant. Note that TINA_w_, which considers species association networks, captures a significantly higher proportion of community variance associated to temperature than Bray-Curtis, a compositional index, in prokaryotes

**Fig. 3:**
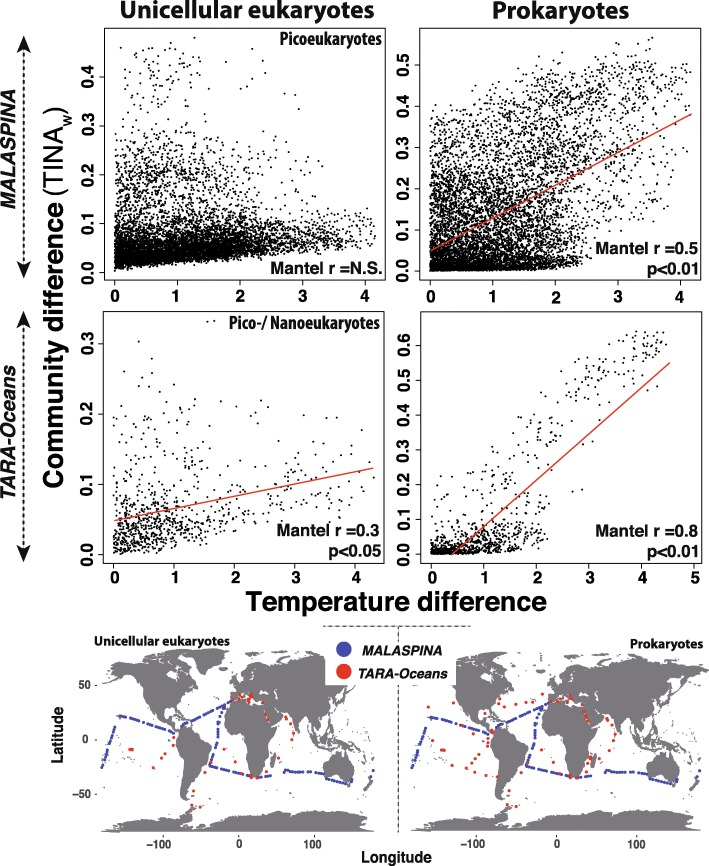
Temperature-driven selection seems to affect species association networks in prokaryotes but not in pico-/nano-eukaryotes. Differences in community composition (as 1-[TINA-weighted] = TINA_w_ dissimilarities) vs. temperature differences (as Euclidean distances based on dimensionless z-scores) for both small unicellular eukaryotes and prokaryotes sampled during the *Malaspina* and *TARA Oceans* expeditions. Note that, in contrast to other indices, TINA_w_ considers species-association patterns (i.e. co-occurrences and co-exclusions ) when estimating β-diversity [26]. NB: While only picoeukaryotes were included in *Malaspina* (cell sizes < 3 μm), *TARA Oceans* data included pico- and nano-eukaryotes (cell sizes < 5 μm). Pico- and nanoeukaryotes from both expeditions (left panels) displayed low or no correlations between TINA_w_ distances and temperature differences (Mantel test results included in the panels). On the contrary, prokaryotes (right panels) displayed high to moderate correlations between TINA_w_ distances and temperature differences. These differences in the correlations are likely due to the wider temperature ranges covered by *TARA Oceans* compared to *Malaspina* (see Discussion)*.* The regression line is shown in red (*Malaspina* microbial eukaryotes N.S., *Malaspina* Prokaryotes *R*^2^ = 0.3, *TARA Oceans* microbial eukaryotes *R*^2^ = 0.1, *TARA Oceans* Prokaryotes *R*^2^ = 0.7; *p* < 0.05). The maps at the bottom indicate the surface stations from the expeditions *Malaspina* (119 stations for both prokaryotes and picoeukaryotes) and *TARA Oceans* (63 stations for prokaryotes and 40 stations for small unicellular eukaryotes) that were used to calculate TINA_w_

We investigated the agents exerting abiotic selection on the tropical and subtropical surface-ocean microbiota by analysing β-diversity together with the environmental variables included in the *Meta*-*119 Malaspina* dataset (temperature (°C), conductivity (S m^−1^), fluorescence, salinity and dissolved oxygen (mL L^−1^)). We used different indices that capture distinct facets of β-diversity (Bray-Curtis, TINA_w_, PINA_w_, gUniFrac; see ‘Methods’ section). Water temperature was the most important driver of selection on prokaryotes (Fig. 2), ranging between 15.7 and 29.3 °C, with a mean of 24.5 °C and a standard deviation of 3.2 °C across the whole *Meta*-*119 Malaspina* dataset (Fig. 1a). Furthermore, water temperature appeared to affect prokaryotic association networks, given that TINA_w_ [26] explained ≈ 50% of community variance (ADONIS *R*^2^) (Fig. 2), while other used β-diversity indices that do not consider species associations explained considerably lower proportions (Fig. 2). In contrast, temperature had limited effects on picoeukaryotic community turnover (Fig. 2). Analyses using both the *Malaspina* and *TARA Oceans* datasets indicated stronger positive correlations between TINA_w_ and water-temperature differences in prokaryotes (Mantel *r* = 0.8–0.5, *p* < 0.01) than in picoeukaryotes [Mantel *r* = 0.3, *p* < 0.05] (Fig. 3). In particular, *TARA Oceans* samples displayed a higher correlation with water temperature than *Malaspina* samples (Fig. 3). Overall, TINA_w_ results indicate that locations with similar temperatures include prokaryotic species that tend to co-occur, with this pattern disappearing as the temperature difference between stations increases. The previous pattern was either weak or non-existent in microbial eukaryotes (Fig. 3).

We expanded the exploration of the role of abiotic selection on microbiota structuring by analysing a larger number of environmental variables (total 17) that were available for only 57 globally distributed *Malaspina* stations (see details in Supplementary Methods, Additional file 6; Figure S4, Additional file 7). Results supported the importance of temperature-driven selection for prokaryotic community structuring (Figure S5, Additional file 8) and indicated that fluorescence (a proxy for Chlorophyll *a* concentration) explained 31% of PINA_w_-based prokaryotic community variance (ADONIS *R*^2^), being non-significant for picoeukaryotes (Figure S5, Additional file 8). The remaining tested abiotic variables explained a minor fraction of community variance, suggesting that abiotic selection, at the whole ocean-microbiota level, operates via few agents, mainly temperature, although we cannot rule out that other unmeasured abiotic variables may also be exerting selection.

The different correlations between temperature and β-diversity as measured by TINA_w_ in prokaryotes and picoeukaryotes suggest that they may feature different species association networks. We found that prokaryotes sampled in both *Malaspina* and *TARA Oceans* were more associated between themselves than protists (Figure S6, Additional file 9; Table S3, Additional file 10; Table S4, Additional file 11; Table S5, Additional file 12). Furthermore, the prokaryotic networks were more modular (in terms of cliques) than the picoeukaryotic counterparts (Table S3, Additional file 10), which may reflect to certain extent, temperature-driven selection [25].

Given that selection exerted by variables that lack phylogenetic signal, typically biotic variables, could inflate estimates of dispersal limitation, we have checked whether the high dispersal limitation we estimated for picoeukaryotes could reflect zooplankton grazing. For that, we have analysed globally distributed surface *TARA Oceans* stations for which we could estimate both the community composition of picoeukaryotes (here defined as the 0.8–5 μm size-fraction; 36 or 38 stations) as well as that of microzooplankton (20–180 μm size-fraction; 36 stations) or mesozooplankton (180–2,000 μm size-fraction; 38 stations) based on 18S-rRNA genes [34]. Analyses considering abiotic (total 6, see Supplementary Methods, Additional file 6) and biotic (estimated zooplankton abundance) variables indicated that micro- and mesozooplankton had a minor influence on picoeukaryotic community structure (≈ 5% of the variance explained, ADONIS *R*^2^). In addition, the correlation between picoeukaryotic and zooplankton β-diversity was either weak (microzooplankton, *ρ* = 0.34) or absent (mesozooplankton) [*p* < 0.01, Mantel tests]. Thus, zooplankton grazing does not appear to influence β-diversity in picoeukaryotes.

### Selection acting on single species

The previous analyses investigated how selection may operate on the entire assemblage of species, without considering the different responses to selection that are expected in individual species. We therefore evaluated the potential action of selection on single species by determining their individual correlations with multiple abiotic environmental variables using the maximal information coefficient (MIC). In the *Malaspina* dataset (Fig. 1a), temperature was the variable with the highest number of associated prokaryotic species (1.7%), representing ≈ 17% of the 16S rRNA gene-sequence abundance, while picoeukaryotic species displayed limited associations with temperature (≈ 0.3% of the species representing ≈ 5% of the 18S rRNA gene-sequence abundance) (Figure S7, Additional file 13). Picoeukaryotic and prokaryotic species were also associated with oxygen, conductivity and salinity (Figure S7, Additional file 13), which covary with temperature. The remaining variables displayed limited associations with individual prokaryotic or picoeukaryotic species (Figure S7, Additional file 13), thus agreeing with our previous results suggesting that abiotic selection on the tropical and subtropical surface-ocean microbiota operates via few variables, with a dominant role for temperature among prokaryotes. Overall, prokaryotes featured proportionally more individual-species associations with environmental parameters than picoeukaryotes (Figure S7, Additional file 13), suggesting that environmental heterogeneity in the tropical and subtropical surface-ocean has a stronger effect on prokaryotic assemblages than on picoeukaryotic counterparts. Analyses of *TARA Oceans* data supported this by indicating that prokaryotic species were associated predominantly with temperature and oxygen in the upper global ocean, while unicellular eukaryotes had weak associations to multiple variables (Table S6, Additional file 14).

### Dispersal

**Fig. 4:**
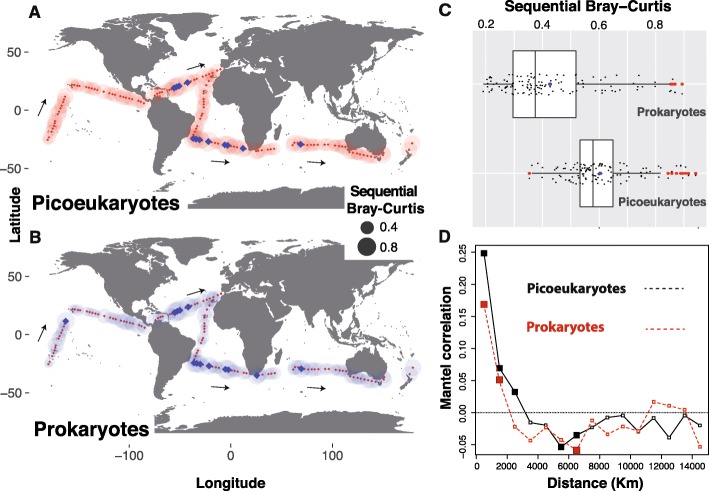
Picoeukaryotic communities display a higher spatial differentiation than prokaryotic counterparts in the tropical-subtropical surface-ocean. **a**–**c** Sequential change in community composition across space (sequential β-diversity). Communities were sampled along the *Malaspina* expedition (**a, b** black arrows), and the composition of each community was compared against its immediate predecessor. In panels **a**, **b**, the size of each bubble represents the Bray-Curtis dissimilarity between a given community and the community sampled previously. Blue squares in panels **a, b** represent the stations where β-diversity displayed abrupt changes (Bray-Curtis values > 0.8 for picoeukaryotes and > 0.7 for prokaryotes). Abrupt changes coincided in a total of 11 out of 14 stations for both picoeukaryotes and prokaryotes, while one station displayed marked changes only for picoeukaryotes and two only for prokaryotes. Panel **c** summarizes the sequential Bray-Curtis values for prokaryotes and picoeukaryotes (Means were significantly different between domains [Wilcoxon text, *p* < 0.05]). Panel **d** indicates the differences in distance-decay between prokaryotes and picoeukaryotes in the tropical and subtropical surface-ocean. Mantel correlograms between geographic distance and β-diversity featuring distance classes of 1000 km for both picoeukaryotes and prokaryotes are shown. Coloured squares indicate statistically significant correlations (*p* < 0.05). Note that β-diversity in picoeukaryotes displayed positive correlations with increasing distances up to ≈ 3000 km, while prokaryotes had positive correlations with distances up to ≈ 2000 km. Correlations tended to be smaller in prokaryotes than in picoeukaryotes, indicating smaller distance decay in the former compared to the latter

Abiotic environmental conditions in adjacent stations over the trajectory of the *Malaspina* cruise, typically separated by 250–500 km, in the tropical and sub-tropical ocean (Fig. 1a) are generally comparable [35]. Therefore, compositional differences between pairs of neighbouring communities could manifest the differential capability of distinct microbial assemblages to disperse. Following these premises, we analysed the change in picoeukaryotic and prokaryotic community composition along the trajectory of the *Malaspina* cruise by comparing each community to the one sampled immediately before in a sequential manner (i.e. sequential β-diversity) (Fig. 4a–c). Both picoeukaryotic and prokaryotic communities displayed variable amounts of sequential β-diversity (Fig. 4a, b), although picoeukaryotes featured, on average, a higher sequential β-diversity than prokaryotes (Fig. 4c). This agrees with the overall mean β-diversity, which was significantly higher for picoeukaryotes than for prokaryotes (Figure S8, Additional file 15). Tests by subsampling the number of picoeukaryotic OTUs_-99%_ to the same number of prokaryotic ones (7025) indicated that different numbers of OTUs_-99%_ in these groups did not affect mean Bray-Curtis estimates of β-diversity displayed in Figure S8, Additional file 15 [36].

When geographic distance covaries with environmental heterogeneity, spatial community variance may be the manifestation of both selection and/or dispersal limitation. β-diversity in picoeukaryotes and prokaryotes displayed positive correlations with geographic distance (i.e. distance decay) predominantly within 1000 km (Fig. 4d). Yet, correlations were weaker in prokaryotes than in picoeukaryotes, pointing to stronger dispersal limitation or selection in the latter. Variance partitioning analyses considering both environmental [temperature (°C), conductivity (S m^−1^), fluorescence, salinity and dissolved oxygen (mL L^−1^)] and geographic variables (ocean basin and subdivisions, as well as Longhurst biogeographic provinces [37], Figure S1, Additional file 1) indicated that in prokaryotes, geographic variables explained most of the variance (24%), while environmental variables explained 10%, and 13% was explained by both variables; 53% of the variance remained unexplained. In contrast, picoeukaryotes displayed non-significant results in the same analyses. Still, after controlling for the effects of the most important environmental variables, Longhurst provinces (but not ocean basins nor subdivisions) accounted for ≈ 20–25% of community variance in both picoeukaryotes and prokaryotes (ADONIS *R*^2^) (Fig. 2). All in all, the previous analyses seem coherent with our quantifications of ecological processes (Fig. 1b), in the sense that they indicate that both selection and dispersal limitation (represented by geographic variables such as distance or ocean provinces), do seem to have a role in the structuring of the surface ocean picoplankton.

Selection and dispersal limitation may operate more strongly in geographic areas that constitute ecological boundaries, leading to abrupt changes in microbiota composition. We identified 14 communities where sequential β-diversity displayed abrupt changes, with 11 of them coinciding for both picoeukaryotes and prokaryotes (Fig. 4a, b). The Local Contributions to Beta Diversity (LCBD) index [38] (Figure S9, Additional file 16) indicated that ≈ 22% of both picoeukaryotic and prokaryotic communities (26 stations each, totaling 36 different stations) contributed the most to the β-diversity, with 16 communities coinciding for both prokaryotes and picoeukaryotes (Figure S9, Additional file 16; Table S7, Additional file 17). In addition, eight of the 36 stations featuring a significant LCBD were also identified as zones of abrupt community change in sequential β-diversity analyses (Table S7, Additional file 17). These zones point to selection or dispersal operating simultaneously and strongly upon both prokaryotic and picoeukaryotic communities in the surface ocean.

## Discussion

Applying an innovative ecological framework [23] allowed us to quantify the mechanisms that shape the tropical and subtropical upper-ocean microbiota. Yet, this approach has limitations (summarised by Zhou and Ning [19]) that need to be considered in the context of our results. *First*, our results represent the overall action of ecological processes at the whole microbiota level, and not their operation on every taxonomic group or lineage (for example, different taxonomic classes may be structured by different processes). In addition, our results reflect the action of ecological mechanisms at the global ocean level, and we expect that other spatial scales (ocean basin for example) may lead to other results. Furthermore, our results provide a snapshot of the importance of ecological processes at the global-ocean scale, and future studies should investigate how the relative importance of these mechanisms change over time [39]. *Second*, the measured ecological mechanisms are associated with the evolutionary diversification that is reflected by the variation in the chosen molecular markers. OTUs_-99%_ and OTUs_-ASVs_ based on the 16S and 18S rRNA genes likely reflect defined species (or gene flow units [40]) or in some cases population variation [32], and therefore, the measured ecological mechanisms in the tropical and subtropical ocean apply to those evolutionary levels. Hence, our results do not reflect the mechanisms shaping intra-population variation or those shaping taxonomic ranks above the species level. Furthermore, our results indicate that delineating OTUs based on sequence clustering (OTUs_-99%_) or sequence variants (OTUs_-ASVs_) can affect measurements of ecological mechanisms, although in our study, main trends were maintained. It could be hypothesized that OTUs_-99%_ and OTUs_-ASVs_ may represent different taxonomic units in prokaryotes or picoeukaryotes, especially if one group was evolving faster than the other. Yet, both prokaryotes and picoeukaryotes show a wide range of evolutionary rates [41, 42], including lineages evolving slow or fast, therefore potential differences in unit definitions associated to different evolutionary rates will likely compensate when analysing complex assemblages of species. *Third*, failure to detect selection could inflate estimates of dispersal limitation. We consider that our estimates indicating substantial dispersal limitation in picoeukaryotes were not inflated, as picoeukaryotes displayed more restricted spatial distributions than prokaryotes and important biotic variables, such as potential zooplankton grazing, did not seem to affect the structure of picoeukaryotic assemblages. Furthermore, another study also suggests that dispersal limitation influences protist distributions in the global ocean [34]. Altogether, the used framework [23] can be considered as a guide that can provide important insights on the ecological mechanisms structuring the global ocean microbiota, while more data (e.g. single nucleotide variants in genes or genomes) and experiments are necessary to understand such mechanisms in further detail.

Our results indicated that the differential action of ecological processes may promote different biogeographic patterns in prokaryotic and picoeukaryotic assemblages in the upper global-ocean. This is consistent with other works using similar approaches to ours indicating that protistan and bacterial assemblages are shaped by different ecological processes [39, 43–45]. In particular, selection, which is known to have an important role in structuring prokaryotic communities [27, 28], explained a higher proportion of community turnover in surface-ocean prokaryotes (≈ 34–27% of the turnover) than in picoeukaryotes (≈ 17–11%). This modest role of selection in structuring the tropical and subtropical sunlit-ocean microbiota is consistent with the moderate environmental gradients characterizing this habitat. In other habitats featuring a higher selective pressure, the role of selection in structuring microbiotas was, as expected, higher [43]. The quantifications of the importance of selection are also associated to the global scale of our survey. Thus, for example, at smaller geographic scales, where dispersal limitation is expected to have a lower impact than at global scales [20], the relative importance of selection could increase. Congruently, in surface waters of the East China Sea, it was found that selection was ~ 40% more important than dispersal limitation in structuring bacterial communities [44], while in our global study, selection and dispersal limitation had a similar importance in structuring prokaryotes. Furthermore, the previous study [44] found that selection was considerably more important than dispersal limitation in structuring communities of microbial eukaryotes. In contrast, our global assessment yields dispersal limitation to be ≈ 5 times more important than selection in structuring picoeukaryotic communities.

We found that heterogeneous selection was more important in structuring picoeukaryotic than prokaryotic communities, while homogeneous selection was more important in structuring prokaryotic than picoeukaryotic communities. This suggests that prokaryotes and picoeukaryotes respond differently to the same environmental heterogeneity, which in the tropical and subtropical surface-ocean would be preventing community divergence in prokaryotes while promoting it in picoeukaryotes. Different adaptations in prokaryotes and picoeukaryotes [9] may determine such contrasting responses to the same environmental heterogeneity. For example, a given environmental heterogeneity could select for a few species featuring wide environmental tolerance or several species that are adapted to narrow environmental conditions.

Several studies have indicated that water temperature is one of the main abiotic variables affecting the structure and diversity of the ocean microbiota [46–52]. Furthermore, temperature is known to structure microbial assemblages in seasonal time-series, pointing also to the importance of this variable at local scales over yearly cycles [53–55]. In our study, the higher correlation between *TARA Oceans* communities with temperature as compared to *Malaspina* (Fig. 3) is coherent with the importance of this variable, as *TARA Oceans* sampled a wider temperature range (range ≈ 0–30 °C, mean ≈ 21 °C, SD ≈ 7 °C) than *Malaspina* (range ≈ 15–30 °C, mean ≈ 24 °C, SD ≈ 3 °C). Furthermore, and consistent with our results, recent global-scale studies reported strong correlations between ocean-microbiota composition (predominantly prokaryotic) and temperature, and weak correlations with nutrients [56, 57]. In sum, the previous agrees with our results indicating that temperature is one of the most important agents exerting abiotic selection on the surface-ocean microbiota, although we cannot rule out the selective action of other unmeasured abiotic factors.

Our analyses also unveiled an additional layer of information by indicating that temperature-driven selection affects prokaryotic taxa co-occurrences, a pattern not observed in picoeukaryotes. Such β-diversity related to species associations is typically not captured by classic compositional indices like Bray-Curtis, possibly due to variations in the relative abundance of the co-occurring species [58]. In contrast to prokaryotes, less is known about the effects of temperature on the community structure of ocean picoeukaryotes, which according to our results are modest. Yet, specific picoeukaryotic lineages, such as MAST-4, do seem to be affected by temperature [59], pointing to taxonomic-group specific responses to selection. One of the possible reasons why picoeukaryotes do not show co-occurrence patterns comparable to those observed in prokaryotes is dispersal limitation, which precludes picoeukaryotic species with similar niches to share the same geographic zone. Overall, our work indicates that species association patterns are informative on the β-diversity of marine prokaryotes, therefore taxa association networks should be contemplated in future analyses of the ocean microbiota.

To what extent dispersal limitation affects the distribution of ocean microbes is a matter of debate. The impact of dispersal limitation is expected to increase with increasing body size [60]; therefore, larger protists are expected to be more limited by dispersal than smaller prokaryotes. Ocean protists seem to follow the previous tenet, as it has been observed that dispersal limitation appears to increase with increasing cell size [34]. Furthermore, in surface open-ocean waters, prokaryotes typically display abundances of 10^6^ cells/mL, while picoeukaryotes normally have abundances of 10^3^ cells/mL [61]. Due to random dispersal alone, the more abundant prokaryotes are expected to be distributed more thoroughly than the less abundant picoeukaryotes [33]. Thus, both cell size and abundance could partially explain our results indicating a higher dispersal limitation in picoeukaryotes than in prokaryotes. Yet, multiple studies of aquatic unicellular eukaryotes point to restricted dispersal [34, 62, 63], while other studies indicate the opposite [59, 64, 65]. This could reflect different dispersal capabilities among unicellular eukaryotes [62, 66] and the generation of dormant cysts in some species [67, 68], which may increase dispersal. Yet, cyst formation has not been reported for picoeukaryotes [9] and this may partially explain their limited dispersal. Regarding prokaryotes, previous studies indicate that dispersal limitation has a modest influence in the structure of marine communities [56, 69, 70], which is coherent with our results. In particular, Louca et al. [71] indicate that there is virtually no dispersal limitation in surface ocean prokaryotes within specific ocean regions, suggesting that the importance of dispersal limitation may increase across large oceanic regions or basins. Nevertheless, dormancy in prokaryotes seems to be more common than in picoeukaryotes [9, 72], and this may allow the former to disperse more thoroughly by reducing their metabolisms when moving through unfavorable habitats [73].

The importance of drift in structuring microbial communities is unclear [27, 74]. Our results, considering both OTUs_-99%_ and OTUs_-ASVs_ indicated that drift has a modest role in structuring picoeukaryotic communities in the tropical and subtropical surface ocean, but a more significant role in structuring prokaryotic counterparts. Another study also found a larger importance of drift in determining the community structure of bacteria when compared with phytoplankton populating freshwater and brackish habitats [75]. In contrast, drift was the prevalent community-structuring mechanism in unicellular eukaryotes populating lakes that feature a strong salinity gradient, having a low importance for the structuring of prokaryotic counterparts [43]; differential adaptations to salinity in protists and prokaryotes may explain these differences [43]. Drift tends to be more important in small populations, which is normally not the case in global ocean microbes. Yet, other random processes could resemble drift in large microbial populations. For example, the arrival of a new bacteriophage may attack abundant bacteria, randomly reshuffling local species abundances.

A decrease in community similarity with increasing geographic distance (distance decay) can be the manifestation of selection and/or dispersal limitation [28]. Distance decay has been evidenced in surface and deep ocean microbiotas [69, 76, 77]. In our study, variance partitioning suggested that both geography (i.e. dispersal limitation) and environmental variation (selection) likely explain distance decay in prokaryotes, with geography having potentially a more important role, which agrees with our ADONIS analyses based on Bray-Curtis and gUnifrac distances (Fig. 2). Interestingly, variance partitioning was not significant in picoeukaryotes, although ADONIS analyses based on Bray-Curtis and gUnifrac distances indicated that geography, and to a lesser extent temperature, would partially explain picoeukaryotic distance decay (Fig. 2).

Overall, provincialism, as measured by Longhurst provinces (Figure S1, Additional file 1), was the most relevant spatial feature for the community structuring of both prokaryotes and picoeukaryotes (Fig. 2). Possibly, this reflects dispersal limitation, as the selective effects of main environmental variables that covary with these provinces were considered in ADONIS analyses. Longhurst provinces may also reflect different water masses or currents that restrict dispersal. Interestingly, a study investigating surface marine bacteria along ≈ 12,000 km in the Atlantic Ocean found that provincialism explained an amount of community variance comparable to our results [69]. Yet, in picoeukaryotes, dispersal limitation may only be partially reflected by provincialism, thus explaining the lack of significance in variance partitioning analyses as well as the differences between the dispersal limitation estimated by provincialism (Fig. 2) and that estimated by ecological processes (Fig. 1b). Alternatively, dispersal limitation in picoeukaryotes may be better reflected by geographic distances between communities, as suggested by sequential Bray-Curtis analyses (Fig. 4c) as well as their stronger distance decay when compared to prokaryotes (Fig. 4d). Furthermore, and consistent with our results, a study of the sunlit global-ocean eukaryotic microbiota indicated that basin, which may be associated to provincialism and dispersal limitation, was one of the most important variables explaining community turnover [34].

In the surface ocean, drastic changes in microbial species composition across space may point to strong changes in abiotic selection (as expected to occur across oceanographic fronts [78, 79]), or high immigration. We identified 14 stations featuring abrupt changes in prokaryotic or picoeukaryotic community composition as well as 36 stations with a “unique” species composition. Some of these areas correspond to nutrient-rich (selection) coastal zones (the South African Atlantic coast and the South Australia Bight) or potential upwelling (dispersal) zones, such as the Equatorial Pacific and Atlantic as well as the Costa Rica Dome. These findings were coherent with spatial abundance distributions (SpAD) of bacterioplankton in the tropical and subtropical surface-ocean [35]. Altogether, the previous suggests strong selective changes or immigration from deep water layers into the surface associated to upwellings, affecting both prokaryotic and picoeukaryotic community structure. Such immigration events into the surface, when random, may partially explain the measured drift.

## Conclusion

Our results indicate that selection, dispersal and drift have different roles in shaping the main components of the picoplankton (prokaryotes and picoeukaryotes) in the tropical and subtropical surface ocean. This highlights the importance of comprehending the characteristics of the different constituents of microbiotas in order to understand their structure. Our results also suggest that the surface ocean picoplankton may not show a single response to global change, and that perhaps prokaryotes will display more pronounced changes in their community structure as a response to temperature increase than picoeukaryotes, considering that temperature seems to affect more prokaryotic than picoeukaryotic assemblages. Future studies on the ocean microbiota should investigate the change in the role of selection, dispersal and drift with ocean scale (from meters to kilometers), depth, latitude and longitude as well as with time, taxonomic ranks (e.g. Class, Family, etc.) and molecular markers that evolve at different rates. Such studies will likely provide a more comprehensive understanding of the underlying mechanisms shaping the ocean microbiota at different evolutionary levels (from lineages to populations) and will also provide insights on the environmental variables that could modify its current configuration.

## Methods

### Sample collection

Surface waters (3 m depth) from a total of 120 globally distributed stations located in the tropical and sub-tropical ocean (Fig. 1a) were sampled as part of the *Malaspina 2010* expedition [30]. Sampling took place between December 2010 and July 2011 and the cruise was organized in a way so that most regions were sampled during similar meteorological seasons. Samples were obtained with a 20 L Niskin bottle deployed simultaneously to a CTD profiler that measured conductivity, temperature, oxygen, fluorescence and turbidity for each sample. About 12 L of seawater were sequentially filtered through a 20 μm nylon mesh, followed by a 3 μm and 0.2 μm polycarbonate filters of 47 mm diameter (Isopore, Millipore, Burlington, MA, USA). Only the smallest size-fraction (0.2–3 μm, here called ‘picoplankton’ [8]) was used in downstream analyses. Samples for inorganic nutrients (NO_3_^−^, NO_2_^−^, PO_4_^3−^, SiO_2_) were collected from the Niskin bottles and measured spectrophotometrically using an Alliance Evolution II autoanalyzer (Frépillon, France) [80]. Chlorophyll measurements were obtained from Estrada et al. [81]. In specific samples, nutrient concentrations were estimated using the World Ocean Database [82] due to issues with the measurements. Since not all environmental parameters were available for all stations, two contextual datasets were generated: *Meta*-*119*, including 119 stations, five environmental parameters and five spatial features **(**all except one station in Fig. 1a) and *Meta*-*57* (Figure S4, Additional file 7), including 57 stations and 17 environmental parameters (the five environmental parameters included in *Meta-119* were considered here as well). See Supplementary Methods, Additional file 6.

### DNA extraction, sequencing and bioinformatics

DNA was extracted using a standard phenol-chloroform protocol [83]. Both the 18S and 16S rRNA-genes were amplified from the same DNA extracts. The hypervariable V4 region of the 18S rRNA gene (≈ 380 bp) was amplified with the primers TAReukFWD1 and TAReukREV3 [84], while the hypervariable V4–V5 (≈ 400 bp) region of the 16S rRNA gene was amplified with the primers 515F-Y-926R [85], which target both Bacteria and Archaea. Amplifications were performed with a QIAGEN HotStar Taq master mix (Qiagen Inc., Valencia, CA, USA). Amplicon libraries were then paired-end sequenced on an *Illumina* (San Diego, CA, USA) MiSeq platform (2 × 250 bp) at the Research and Testing Laboratory facility (http://www.researchandtesting.com/). See additional details on gene amplification and sequencing in Supplementary Methods, Additional file 6.

Reads were processed following and in-house protocol [86]. Briefly, raw reads were corrected using BayesHammer [87] following Schirmer et al. [88]. Corrected paired-end reads were subsequently merged with PEAR [89] and sequences longer than 200 bp were quality-checked (maximum expected errors [maxEE] = 0.5) and de-replicated using USEARCH V8.1.1756 [90]. Operational taxonomic units (OTUs) were delineated at 99% similarity using UPARSE V8.1.1756 [91], producing 42,505 picoeukaryotic and 10,158 prokaryotic OTUs_-99%_. Taxonomic assignment of OTUs_-99%_ was generated by BLASTing OTU-representative sequences against different reference databases. BLAST hits were filtered prior to taxonomy assignment using an in-house python script, considering a percentage of identity > 90%, a coverage > 70%, a minimum alignment length of 200 bp and an e-value < 0.00001. Metazoan, Streptophyta, nucleomorphs, Chloroplast and mitochondrial OTUs were removed from the OTUs_-99%_ tables. See Supplementary Methods, Additional file 6 and Table S8, Additional file 18.

Additionally, to investigate the effects of clustering on the estimation of ecological mechanisms (Fig. 1b), we determined OTUs as amplicon sequence variants (ASVs) using DADA2 [92]. For the 18S, we trimmed the forward reads at 240 bp and the reverse reads at 180 bp, while for the 16S, forward reads were trimmed at 220 bp and reverse reads at 200 bp. Then, for the 18S, the maximum number of expected errors (maxEE) was set to 7 and 8 for the forward and reverse reads respectively, while for the 16S, the maxEE was set to 2 for the forward reads and to 4 for the reverse reads. Error rates were estimated with DADA2 for both the 18S and 16S and used to delineate OTUs_-ASVs_ (see additional details in Supplementary Methods, Additional file 6). A total of 21,970 and 6196 OTUs_-ASVs_ were delineated for the 18S and 16S respectively.

OTUs_-ASVs_ were assigned taxonomy using the naïve Bayesian classifier method [93] together with the SILVA version 132 [94] database as implemented in DADA2. Eukaryotic OTUs_-ASVs_ were also BLASTed [95] against the Protist Ribosomal Reference database (PR^2^, version 4.11.1 [96];). Streptophyta, Metazoa, nucleomorphs, chloroplasts and mitochondria were removed from OTUs_-ASVs_ tables. Tables of OTUs_-ASVs_ were rarefied to 20,000 reads per sample with the function *rrarefy* in Vegan. Only OTUs_-ASVs_ with abundances > 100 reads were used for the calculation of ecological mechanisms (Fig. 1b).

We tested the similarity of OTUs_-99%_ and OTUs_-ASVs_ between themselves as well as against a reference database (SILVA v132) in order to determine whether there were differences in the OTUs delineated by UPARSE or DADA2. Comparisons were run using BLAST, and only best hits featuring a sequence similarity > 90%, e-value < 0.001, query coverage > 60% and alignment length > 200 bp were considered. For the 16S, OTUs_-ASVs_ vs. OTUs_-99%_ displayed a 99.0% (SD = 2.0%) mean similarity, while for the 18S, both types of OTUs had 99.3% (SD = 1.4%) mean similarity. Furthermore, for the 16S, the mean similarity to SILVA reference sequences was 98.8% (SD = 1.5%) for OTUs_-99%_ and 98.5% (SD = 2.2%) for OTUs_-ASVs_. In turn, for the 18S, the mean similarity against SILVA v132 was 97.8% (SD = 2.0%) for OTUs_-99%_ and 97.2 % (SD = 2.5%) for OTUs_-ASVs_. In sum, these analyses indicate a high similarity between OTUs_-ASVs_ and OTUs_-99%_, both having also comparable levels of similarity to reference sequences, which indicates that the two approaches to delineate OTUs (i.e. UPARSE vs. DADA2) have similar error-rates.

We used publicly available data from the *TARA Oceans* global expedition [31] in multiple analyses. This expedition took place between September 2009 and March 2012, and includes samples from the same hemisphere during different meteorological seasons. Due to the nature of the *TARA Oceans* dataset, we did not perform all the analyses that were run for the *Malaspina* dataset. Specifically, short V9 18S rRNA-gene reads or 16S rRNA-gene miTags [97] from *TARA Oceans* precluded robust phylogenetic reconstructions, which instead were possible with the longer reads produced for *Malaspina.* We used data from *TARA Oceans* surface (≈ 5 m depth) stations only, including 41 samples (40 stations) for pico-nano eukaryotes (0.22–3 μm [one sample] and 0.8–5 μm [40 samples]; 18S-V9 rRNA gene amplicon data) [34] as well as 63 stations for prokaryotes (picoplankton, 0.22–3 μm [45 samples] and 0.22–1.6 μm [18 samples]; 16S rRNA genes, miTags) [56].

### General analyses and phylogenetic inferences

Tables including OTUs_-99%_ were sub-sampled to 4060 reads per sample using *rrarefy* in *Vegan* [98], resulting in sub-sampled tables containing 18,775 picoeukaryotic and 7025 prokaryotic OTUs. OTUs_-99%_ with mean relative abundances > 0.1% or < 0.001% were defined as regionally abundant or rare respectively [99]. Phylogenetic trees were constructed by aligning 16S or 18S OTUs_-99%_ representative sequences or OTUs_-ASVs_ against an aligned SILVA [94] template using mothur [100]. Afterwards, poorly aligned regions or sequences were removed using trimAl [101]. Phylogenetic trees were inferred using FastTree v2.1.9 [102]. Most analyses were performed in the R statistical environment [103] using *APE* [104], *ggplot2* [105], *gUniFrac* [106], *Maps*, *Mapplots*, *Picante* [107] and *Vegan*. The *Vegan* function *adonis* and *adonis2* were used to investigate the amount of variance in community composition explained by environmental or geographic variables. Variance partitioning analyses were run with *varpart* in *Vegan* and tested for significance with ANOVA. Distance decay, which refers to the decrease in microbial community similarity as geographic distance between communities increases, was investigated in R using Mantel correlograms between geographic distance and β-diversity, considering distance classes of 1000 km. Local contributions to beta diversity (LCBD) [38], which indicates the degree of uniqueness of each community in terms of its species composition, was measured with *adespatial* [108]. See Supplementary Methods, Additional file 6.

### Quantification of selection, dispersal and drift

These processes were quantified using an approach that relies on null models, consisting of two main sequential steps: the first uses OTU phylogenetic turnover to infer the action of selection and the second uses OTU compositional turnover to infer the action of dispersal and drift [23]. The action of selection, dispersal and drift was quantified using both OTUs_-99%_ and OTUs_-ASVs_. In order to determine the action of selection using phylogenetic turnover, we first checked whether habitat preferences of phylogenetically closely related taxa (according to the 16S and 18S rRNA-genes) were more similar to each other than to those of more distantly related taxa, what is known as *phylogenetic signal* [109, 110]. We tested for phylogenetic signal using temperature and fluorescence, which were the two variables that explained the highest fraction of community variance. We detected phylogenetic signal at relatively short phylogenetic distances (Figure S10, Additional file 19; Figure S11, Additional file 20), which is coherent with previous work [23, 111, 112]. We measured phylogenetic turnover using the abundance-weighted β-mean nearest taxon distance (βMNTD) metric [19, 23], which quantifies the mean phylogenetic distances between the evolutionary-closest OTUs in two communities. βMNTD values can be larger, smaller or equal to the values expected when selection is not affecting community turnover (that is, expected by chance). βMNTD values higher than expected by chance indicate that communities experience heterogeneous selection [19]. In contrast, βMNTD values which are lower than expected by chance indicate that communities experience homogeneous selection. Null models included 999 randomizations [23]. Differences between the observed βMNTD and the mean of the null distribution are denoted as β-Nearest Taxon Index (βNTI), with |βNTI| > 2 being considered as significant departures from random phylogenetic turnover, pointing to the action of selection.

The second step uses OTU turnover to calculate whether the β-diversity of communities not structured by selection could be generated by drift (i.e. chance) or dispersal. We calculated the Raup-Crick metric [113] using Bray-Curtis dissimilarities (hereafter RC_bray_) [23]. RC_bray_ compares the measured β-diversity against the β-diversity that would be obtained under random community assembly (drift); randomizations were run 9999 times. RC_bray_ values between − 0.95 and + 0.95 point to a community assembly governed by drift. On the contrary, RC_bray_ values > + 0.95 or < − 0.95 indicate that community turnover is driven by dispersal limitation or homogenising dispersal respectively [113]. See Supplementary Methods, Additional file 6.

### Estimation of interaction-adjusted indices

Taxa INteraction-Adjusted (TINA) and Phylogenetic INteraction Adjusted (PINA) indices were estimated following Schmidt et al. [26]. TINA is based on taxa co-occurrences while PINA considers phylogenetic similarities. TINA quantifies β-diversity as the average association strength between all taxa in different samples. Thus, communities which are identical or include taxa that are perfectly associated will give a TINA value of 1. TINA values will approach 0.5 in communities sharing no taxa or having neutral associations, and approach 0 if taxa display high avoidance. Dissimilarity matrices were generated as 1-TINA and used in downstream analyses (e.g. Fig. 3). Full picoeukaryotic and prokaryotic subsampled OTU_-99%_ tables were used to calculate the abundance-weighted TINA_w_ and PINA_w_. TINA_w_ was calculated using picoeukaryotic and prokaryotic data from 119 *Malaspina* surface stations (most stations in Fig. 1a). In addition, TINA_w_ was calculated using data from *TARA Oceans*, including 63 surface stations for prokaryotes and 40 surface station for small unicellular eukaryotes (Fig. 3).

### Associations between taxa and environmental parameters

We analysed whether OTUs_-99%_ displayed associations with environmental variables and between themselves. Firstly, we used the maximal information coefficient (MIC), which captures diverse relationships between two pairs of variables [114]. The *Malaspina* dataset consisted of 119 stations and 17 environmental variables. In the *TARA Oceans* dataset, prokaryotes were analysed across 63 surface stations (including eight environmental variables), while microbial eukaryotes were analysed across 40 surface stations (including six environmental variables) (see Supplementary Methods, Additional file 6). In both datasets, MIC analyses were run using CV = 0.5, *B* = 0.6 and statistically significant relationships with MIC ≥ 0.4 (*Malaspina*) or MIC ≥ 0.5 (*TARA Oceans*) were considered (MIC thresholds were adjusted to the characteristics of the datasets). MIC significance was assessed using precomputed *p* values [114]. Secondly, we constructed association networks with the *Malaspina* dataset considering OTUs_-99%_ with > 100 reads using SparCC [115] as implemented in FastSpar [116]. To determine correlations, FastSpar was run with 1000 iterations, including 1000 bootstraps to infer *p* values. We used OTUs_-99%_ associations with absolute correlation scores > 0.3 and *p* value < 0.01. Networks were visualized and analysed with Cytoscape [117] and igraph [118].

## Supplementary information

**Additional file 1: Figure S1.** Position of the 120 analysed *Malaspina-2010* stations in the context of the Longhurst biogeographic provinces [37].

**Additional file 2: Figure S2.** Bray Curtis and gUniFrac distances between picoeukaryotes and prokaryotes from the *Malaspina* dataset. Regression (blue) and 0:1 (red) lines are indicated.

**Additional file 3: Table S1. ** Regionally abundant or rare prokaryotic and picoeukaryotic OTUs_-99%_ from the Malaspina dataset.

**Additional file 4: Figure S3.** OTUs_-99%_ mean relative abundance (i.e. regional abundance) vs. occurrence (i.e. number of samples in which each OTUs_-99%_ is present) for the *Malaspina* dataset. The red and black horizontal lines indicate percentages of occurrences of 80% and 20% respectively. Cosmopolitan OTUs were considered as those with a percentage of occurrence >80%, while restricted OTUs were those with a percentage of occurrence <20% (see Table S2, Additional file 5). Blue and green vertical lines indicate regional abundances above and below which OTUs are considered regionally abundant (>0.1%) or rare (<0.001%) respectively.

**Additional file 5: Table S2.** OTUs_-99%_ displaying Cosmopolitan, Intermediate and Restricted distributions in the Malaspina dataset.

**Additional file 6:** Supplementary Methods.

**Additional file 7: Figure S4.** The 57 *Malaspina* stations for which 17 environmental parameters were available (*Meta-57* dataset).

**Additional file 8: Figure S5.** Percentage of variance in Picoeukaryotic and Prokaryotic community composition (ADONIS *R*^2^) explained by water temperature and fluorescence when using different β-diversity metrics. Figure based on the *Malaspina Meta-57* dataset.

**Additional file 9: Figure S6.** Species association networks for the tropical and subtropical surface-ocean microbiota as inferred from the *Malaspina* dataset. Left-hand side: Association networks of picoeukaryotes and prokaryotes considering positive (red) and negative (blue) correlations in panels A) [Eukaryotic Network (+-e)] and B) [Prokaryotic Network (+-e)], and only positive correlations in C) [Eukaryotic Network (+e)] and D) [Prokaryotic Network (+e)]. On the right-hand side, we present an alternative visualization of the network as well as the following network characteristics: number of nodes (n), number of edges with positive correlation (+e) and negative correlation (-e), average degree (avg. d), average path length (avg. l), global transitivity (t), number of modules with at least 3 nodes (m) and the number of nodes in each of those modules (sizes: n). The smaller network visualization on the right-hand side groups the nodes according to the modules. The colors of nodes in Left- and Right-hand side networks indicate the modules to which they belong (NB: colors in panels A, B, C & D are independent of each other).

**Additional file 10: Table S3.** Summary of association networks from the *Malaspina* dataset based on SparCC.

**Additional file 11: Table S4.** Summary of significant OTUs_-99%_ associations using MIC for the *Malaspina* dataset.

**Additional file 12: Table S5.** Summary of significant OTUs_-99%_ associations  for the *TARA Oceans* dataset based on MIC.

**Additional file 13: Figure S7.** Percentage of OTUs_-99%_ significantly associated to different environmental variables (MIC > 0.4) [left] and their corresponding contribution to total sequence abundance (i.e. percentage of reads) [right] in the *Malaspina* dataset. NB: Temperature, Oxygen, Conductivity and Salinity are correlated. OTUs can be associated to more than one variable.

**Additional file 14: Table S6.** Significant MIC associations (MIC > 0.5) between OTUs and environmental parameters in the *TARA Oceans* dataset.

**Additional file 15: Figure S8.** Bray-Curtis dissimilarities and gUniFrac distances in Prokaryotes and Picoeukaryotes from the *Malaspina* dataset. In both cases, mean differences were significant (Wilcoxon text, p<0.05). Prokaryotes (Bray Curtis mean=0.61, SD=0.19; gUniFrac mean=0.30, SD=0.07); Picoeukaryotes (Bray Curtis mean=0.74, SD=0.08; gUniFrac mean=0.50, SD=0.06).

**Additional file 16: Figure S9.** Stations (total 36) from the *Malaspina* dataset featuring a comparatively large contribution to the overall β-diversity (LCBD = Local Contributions to Beta Diversity [38]; p<0.05).

**Additional file 17: Table S7.** The 36 *Malaspina* stations (out of 120) featuring significant (*p* < 0.05) Local Contributions to Beta Diversity (LCBD) in prokaryotes and/or picoeukaryotes.

**Additional file 18: Table S8.***Malaspina* eukaryotic and prokaryotic reads and OTUs_-99%_ analysed during different steps of our in-house workflow.

**Additional file 19: Figure S10.** Phylogenetic signal was detected across short phylogenetic distances for both the 16S and 18S rRNA-gene markers as indicated by phylogenetic mantel correlograms (*Malaspina* dataset). Phylogenetic signal was tested using temperature and fluorescence, the two variables that explain the highest fraction of community variance. Solid and open squares indicate significant and nonsignificant (using p=0.05) correlations respectively between environmental similarity (in terms of temperature and fluorescence) and phylogenetic relatedness. Correlations that are significantly positive indicate that the phylogenetic distance between OTUs_-99%_ increases as environmental similarity decreases for the phylogenetic range being analysed. Phylogenetic distances were measured as abundance-weighted β-Mean Nearest Taxon Distances (βMNTD).

**Additional file 20: Figure S11.** Same as Figure S10, Additional file 19 but using OTUs_-ASVs_. Solid and open squares indicate significant and nonsignificant (using p=0.05) correlations respectively between environmental similarity (in terms of temperature and fluorescence) and phylogenetic relatedness. Correlations that are significantly positive indicate that the phylogenetic distance between OTUs_-ASVs_ increases as environmental similarity decreases for the phylogenetic range being analysed. Phylogenetic distances were measured as abundance-weighted β-Mean Nearest Taxon Distances (βMNTD).

## Data Availability

DNA sequences and metadata from the *Malaspina* expedition are publicly available at the European Nucleotide Archive (http://www.ebi.ac.uk/ena; accession numbers PRJEB23913 [18S rRNA genes] & PRJEB25224 [16S rRNA genes]). The data used from *TARA Oceans* is publicly available through *Pangaea* (10.1594/PANGAEA.873275) as well as in (http://ocean-microbiome.embl.de/companion.html) [34, 56]. The code for generating OTU_-99%_ tables is available in: 10.5281/zenodo.259579. R-Scripts for calculating the β-Nearest Taxon Index and the Raup-Crick metric are available in https://github.com/stegen/Stegen_etal_ISME_2013. The code for calculating the TINA and PINA indices is available in https://github.com/defleury/Schmidt_et_al_2016_community_similarity, while the code for calculating MIC is available at http://www.exploredata.net. All used R packages as well as other software are cited in ‘Methods’ section.

## References

[1] Falkowski P (2012). The power of plankton. Nature..

[2] Falkowski PG, Fenchel T, Delong EF (2008). The microbial engines that drive Earth's biogeochemical cycles. Science..

[3] Jardillier L, Zubkov MV, Pearman J, Scanlan DJ (2010). Significant CO_2_ fixation by small prymnesiophytes in the subtropical and tropical northeast Atlantic Ocean. ISME J..

[4] Li WKW (1994). Primary production of prochlorophytes, cyanobacteria, and eucaryotic ultraphytoplankton: Measurements from flow cytometric sorting. Limnol Oceanography..

[5] Worden AZ, Follows MJ, Giovannoni SJ, Wilken S, Zimmerman AE, Keeling PJ (2015). Rethinking the marine carbon cycle: factoring in the multifarious lifestyles of microbes. Science..

[6] Field CB, Behrenfeld MJ, Randerson JT, Falkowski P (1998). Primary production of the biosphere: integrating terrestrial and oceanic components. Science..

[7] del Giorgio PA, Duarte CM (2002). Respiration in the open ocean. Nature..

[8] Massana R (2011). Eukaryotic picoplankton in surface oceans. Annual review of microbiology..

[9] Massana R, Logares R (2013). Eukaryotic versus prokaryotic marine picoplankton ecology. Environ Microbiol..

[10] Picoeukaryotes MR, Schaechter M (2009). *Encyclopedia of Microbiology*.

[11] Seymour JR, Amin SA, Raina JB, Stocker R (2017). Zooming in on the phycosphere: the ecological interface for phytoplankton-bacteria relationships. Nat Microbiol..

[12] Jürgens K, Massana R. Protistan grazing on marine bacterioplankton, 2nd edn. Hoboken, New Jersey: Wiley-Blackwell; 2008.

[13] Hellweger FL, van Sebille E, Fredrick ND (2014). Biogeographic patterns in ocean microbes emerge in a neutral agent-based model. Science..

[14] Gibbons SM, Caporaso JG, Pirrung M, Field D, Knight R, Gilbert JA (2013). Evidence for a persistent microbial seed bank throughout the global ocean. Proc Natl Acad Sci U S A..

[15] Nemergut DR, Schmidt SK, Fukami T, O'Neill SP, Bilinski TM, Stanish LF, Knelman JE, Darcy JL, Lynch RC, Wickey P (2013). Patterns and processes of microbial community assembly. Microbiol Mol Biol Rev..

[16] Leibold MA, Chase JM, Ernest SK (2017). Community assembly and the functioning of ecosystems: how metacommunity processes alter ecosystems attributes. Ecology..

[17] Mori AS, Isbell F, Seidl R (2018). Beta-diversity, community assembly, and ecosystem functioning. Trends Ecol Evol..

[18] Vellend M (2016). The theory of ecological communities.

[19] Zhou J, Ning D (2017). Stochastic community assembly: does it matter in microbial ecology?. Microbiol Mol Biol Rev..

[20] Heino J, Melo AS, Siqueira T, Soininen J, Valanko S, Bini LM (2015). Metacommunity organisation, spatial extent and dispersal in aquatic systems: patterns, processes and prospects. Freshwater Biology..

[21] Hubbell SP (2001). A unified neutral theory of biodiversity and biogeography.

[22] Holyoak M, Leibold MA, Holt RD (2005). Metacommunities: spatial dynamics and ecological communities.

[23] Stegen JC, Lin X, Fredrickson JK, Chen X, Kennedy DW, Murray CJ, Rockhold ML, Konopka A (2013). Quantifying community assembly processes and identifying features that impose them. ISME J..

[24] Magurran AE, McGill BJ. Biological diversity: frontiers in measurements and assessment: Oxford University Press; 2011.

[25] Röttjers L, Faust K (2018). From hairballs to hypotheses—biological insights from microbial networks. FEMS Microbiol Rev..

[26] Schmidt TS, Matias Rodrigues JF, von Mering C (2017). A family of interaction-adjusted indices of community similarity. ISME J..

[27] Lindström ES, Langenheder S (2012). Local and regional factors influencing bacterial community assembly. Environ Microbiol Rep..

[28] Hanson CA, Fuhrman JA, Horner-Devine MC, Martiny JB (2012). Beyond biogeographic patterns: processes shaping the microbial landscape. Nature reviews Microbiology..

[29] Fisher CK, Mehta P (2014). The transition between the niche and neutral regimes in ecology. Proc Natl Acad Sci U S A..

[30] Duarte CM (2015). Seafaring in the 21St Century: The Malaspina 2010 circumnavigation expedition. Limnol Oceanography Bull.

[31] Karsenti E, Acinas SG, Bork P, Bowler C, De Vargas C, Raes J, Sullivan M, Arendt D, Benzoni F, Claverie JM (2011). A holistic approach to marine eco-systems biology. PLoS biology..

[32] Caron DA, Hu SK (2019). Are we overestimating protistan diversity in nature?. Trends Microbiol..

[33] Gaston KJ, Blackburn TM, Greenwood JJD, Gregory RD, Quinn RM, Lawton JH (2000). Abundance–occupancy relationships. J Appl Ecol.

[34] de Vargas C, Audic S, Henry N, Decelle J, Mahe F, Logares R, Lara E, Berney C, Le Bescot N, Probert I (2015). Eukaryotic plankton diversity in the sunlit ocean. Science..

[35] Ruiz-Gonzalez C, Logares R, Sebastian M, Mestre M, Rodriguez-Martinez R, Gali M, Sala MM, Acinas SG, Duarte CM, Gasol JM (2019). Higher contribution of globally rare bacterial taxa reflects environmental transitions across the surface ocean. Mol Ecol..

[36] Kraft NJ, Comita LS, Chase JM, Sanders NJ, Swenson NG, Stegen JC, Vellend M, Boyle B, Anderson MJ, Crist TO (2011). Disentangling the drivers of beta diversity along latitudinal and elevational gradients. Science..

[37] Longhurst AR. Ecological geography of the sea: Academic Press; 2007.

[38] Legendre P, De Caceres M (2013). Beta diversity as the variance of community data: dissimilarity coefficients and partitioning. Ecol Lett..

[39] Vass M, Székely AJ, Lindström ES, Langenheder S. Using null models to compare bacterial and microeukaryotic metacommunity assembly under shifting environmental conditions. Sci Rep. 2020;10(1):2455. 10.1038/s41598-020-59182-1 PMC7016149 32051469

[40] Arevalo P, VanInsberghe D, Elsherbini J, Gore J, Polz MF (2019). A reverse ecology approach based on a biological definition of microbial populations. Cell.

[41] Pernice MC, Logares R, Guillou L, Massana R (2013). General patterns of diversity in major marine microeukaryote lineages. PLoS One..

[42] Duchêne S, Holt KE, Weill F-X, Le Hello S, Hawkey J, Edwards DJ, Fourment M, Holmes EC (2016). Genome-scale rates of evolutionary change in bacteria. Microb Genom..

[43] Logares R, Tesson SVM, Canback B, Pontarp M, Hedlund K, Rengefors K (2018). Contrasting prevalence of selection and drift in the community structuring of bacteria and microbial eukaryotes. Environ Microbiol..

[44] Wu W, Lu HP, Sastri A, Yeh YC, Gong GC, Chou WC, Hsieh CH (2018). Contrasting the relative importance of species sorting and dispersal limitation in shaping marine bacterial versus protist communities. ISME J..

[45] Brislawn CJ, Graham EB, Dana K, Ihardt P, Fansler SJ, Chrisler WB, Cliff JB, Stegen JC, Moran JJ, Bernstein HC (2019). Forfeiting the priority effect: turnover defines biofilm community succession. ISME J..

[46] Sul WJ, Oliver TA, Ducklow HW, Amaral-Zettler LA, Sogin ML (2013). Marine bacteria exhibit a bipolar distribution. Proc Natl Acad Sci U S A..

[47] Fuhrman JA, Steele JA, Hewson I, Schwalbach MS, Brown MV, Green JL, Brown JH (2008). A latitudinal diversity gradient in planktonic marine bacteria. Proc Natl Acad Sci U S A..

[48] Pommier T, Canbäck B, Riemann L, Boström H, Simu K, Lundberg P, Tunlid A, Hagström Å (2007). Global patterns of diversity and community structure in marine bacterioplankton. Mol Ecol.

[49] Rutherford S, D'Hondt S, Prell W (1999). Environmental controls on the geographic distribution of zooplankton diversity. Nature..

[50] Johnson ZI, Zinser ER, Coe A, McNulty NP, Woodward EMS, Chisholm SW (2006). Niche partitioning among Prochlorococcus ecotypes along ocean-scale environmental gradients. Science..

[51] Raes J, Letunic I, Yamada T, Jensen LJ, Bork P (2011). Toward molecular trait-based ecology through integration of biogeochemical, geographical and metagenomic data. Mol Syst Biol.

[52] Ibarbalz FM, Henry N, Brandao MC, Martini S, Busseni G, Byrne H, Coelho LP, Endo H, Gasol JM, Gregory AC (2019). Global Trends in Marine Plankton Diversity across Kingdoms of Life. Cell..

[53] Giner CR, Balague V, Krabberod AK, Ferrera I, Rene A, Garces E, Gasol JM, Logares R, Massana R (2019). Quantifying long-term recurrence in planktonic microbial eukaryotes. Mol Ecol..

[54] Lambert S, Tragin M, Lozano J-C, Ghiglione J-F, Vaulot D, Bouget F-Y, Galand PE (2019). Rhythmicity of coastal marine picoeukaryotes, bacteria and archaea despite irregular environmental perturbations. ISME J..

[55] Bunse C, Pinhassi J (2017). Marine bacterioplankton seasonal succession dynamics. Trends Microbiol..

[56] Sunagawa S, Coelho LP, Chaffron S, Kultima JR, Labadie K, Salazar G, Djahanschiri B, Zeller G, Mende DR, Alberti A (2015). Structure and function of the global ocean microbiome. Science..

[57] Salazar G, Paoli L, Alberti A, Huerta-Cepas J, Ruscheweyh HJ, Cuenca M, Field CM, Coelho LP, Cruaud C, Engelen S (2019). Gene expression changes and community turnover differentially shape the global ocean metatranscriptome. Cell..

[58] Chase JM (2003). Community assembly: when should history matter?. Oecologia..

[59] Rodriguez-Martinez R, Rocap G, Salazar G, Massana R (2013). Biogeography of the uncultured marine picoeukaryote MAST-4: temperature-driven distribution patterns. ISME J..

[60] De Bie T, De Meester L, Brendonck L, Martens K, Goddeeris B, Ercken D, Hampel H, Denys L, Vanhecke L, Van der Gucht K (2012). Body size and dispersal mode as key traits determining metacommunity structure of aquatic organisms. Ecology Letters..

[61] Kirchman DL (2008). Microbial Ecology of the Oceans.

[62] Foissner W (2006). Biogeography and dispersal of micro-organisms: a review emphasizing protists. Acta Protozoologica..

[63] Casteleyn G, Leliaert F, Backeljau T, Debeer AE, Kotaki Y, Rhodes L, Lundholm N, Sabbe K, Vyverman W (2010). Limits to gene flow in a cosmopolitan marine planktonic diatom. Proc Natl Acad Sci U S A..

[64] Cermeno P, Falkowski PG (2009). Controls on diatom biogeography in the ocean. Science..

[65] Whittaker KA, Rynearson TA (2017). Evidence for environmental and ecological selection in a microbe with no geographic limits to gene flow. Proc Natl Acad Sci U S A..

[66] Bass D, Richards TA, Matthai L, Marsh V, Cavalier-Smith T (2007). DNA evidence for global dispersal and probable endemicity of protozoa. BMC Evol Biol..

[67] Lewis J, Harris ASD, Jones KJ, Edmonds RL (1999). Long-term survival of marine planktonic diatoms and dinoflagellates in stored sediment samples. J Plankton Res..

[68] Billard C, Inouye I, Thierstein HR, Young JR (2004). What is new in coccolithophore biology?. *Coccolithophores: From Molecular Processes to Global Impact*.

[69] Milici M, Tomasch J, Wos-Oxley ML, Decelle J, Jauregui R, Wang H, Deng ZL, Plumeier I, Giebel HA, Badewien TH (2016). Bacterioplankton biogeography of the Atlantic Ocean: a case study of the distance-decay relationship. Front Microbiol..

[70] Sintes E, De Corte D, Ouillon N, Herndl GJ (2015). Macroecological patterns of archaeal ammonia oxidizers in the Atlantic Ocean. Mol Ecol..

[71] Louca S, Parfrey LW, Doebeli M (2016). Decoupling function and taxonomy in the global ocean microbiome. Science..

[72] Jones SE, Lennon JT (2010). Dormancy contributes to the maintenance of microbial diversity. Proc Natl Acad Sci U S A..

[73] Locey KJ (2010). Synthesizing traditional biogeography with microbial ecology: the importance of dormancy. J Biogeography..

[74] Louca S, Polz MF, Mazel F, Albright MBN, Huber JA, O’Connor MI, Ackermann M, Hahn AS, Srivastava DS, Crowe SA (2018). Function and functional redundancy in microbial systems. Nat Ecol Evol.

[75] Östman Ö, Drakare S, Kritzberg ES, Langenheder S, Logue JB, Lindström ES (2010). Regional invariance among microbial communities. Ecology letters..

[76] Salazar G, Cornejo-Castillo FM, Benitez-Barrios V, Fraile-Nuez E, Alvarez-Salgado XA, Duarte CM, Gasol JM, Acinas SG (2016). Global diversity and biogeography of deep-sea pelagic prokaryotes. ISME J..

[77] Zinger L, Boetius A, Ramette A (2014). Bacterial taxa–area and distance–decay relationships in marine environments. Mol Ecol..

[78] Díez B, Massana R, Estrada M, Pedrós-Alió C (2004). Distribution of eukaryotic picoplankton assemblages across hydrographic fronts in the Southern Ocean, studied by denaturing gradient gel electrophoresis. Limnol Oceanography..

[79] Flaviani F, Schroeder D, Lebret K, Balestreri C, Schroeder J, Moore K, Paszkiewicz K, Pfaff M, Rybicki E. Distinct oceanic microbiomes (from viruses to protists) found either side of the Antarctic Polar Front. Front Microbiol. 2018;9. 10.3389/fmicb.2018.01474 PMC6056678 30065704

[80] Grasshoff K, Ehrhardt M, Kremling K. Methods of seawater analysis. Weinheim: Verlag Chemie; 1983.

[81] Estrada M, Delgado M, Blasco D, Latasa M, Cabello AM, Benitez-Barrios V, Fraile-Nuez E, Mozetic P, Vidal M (2016). Phytoplankton across tropical and subtropical regions of the Atlantic, Indian and Pacific Oceans. PLoS One.

[82] Boyer TP, Antonov JI, Baranova OK, Coleman C, Garcia HE, Grodsky A, Johnson DR, Locarnini RA, Mishonov AV, O'Brien TD, Levitus S, Mishonov A (2013). World Ocean Database 2013. In: *NOAA Atlas NESDIS 72*.

[83] Massana R, Murray AE, Preston CM, DeLong EF (1997). Vertical distribution and phylogenetic characterization of marine planktonic Archaea in the Santa Barbara Channel. Appl Environ Microbiol..

[84] Stoeck T, Bass D, Nebel M, Christen R, Jones MD, Breiner HW, Richards TA (2010). Multiple marker parallel tag environmental DNA sequencing reveals a highly complex eukaryotic community in marine anoxic water. Mol Ecol..

[85] Parada AE, Needham DM, Fuhrman JA (2016). Every base matters: assessing small subunit rRNA primers for marine microbiomes with mock communities, time series and global field samples. Environ Microbiol..

[86] Logares R. Workflow for Analysing MiSeq Amplicons based on Uparse v1.5. In*.*: 10.5281/zenodo.259579; 2017.

[87] Nikolenko SI, Korobeynikov AI, Alekseyev MA. BayesHammer: Bayesian clustering for error correction in single-cell sequencing. BMC Genomics. 2013; 14 Suppl 1:S7. 10.1186/1471-2164-14-S1-S7 PMC3549815 23368723

[88] Schirmer M, Ijaz UZ, D'Amore R, Hall N, Sloan WT, Quince C (2015). Insight into biases and sequencing errors for amplicon sequencing with the Illumina MiSeq platform. Nucleic Acids Res..

[89] Zhang J, Kobert K, Flouri T, Stamatakis A (2014). PEAR: a fast and accurate Illumina Paired-End reAd mergeR. Bioinformatics..

[90] Edgar RC (2010). Search and clustering orders of magnitude faster than BLAST. Bioinformatics..

[91] Edgar RC (2013). UPARSE: highly accurate OTU sequences from microbial amplicon reads. Nat Methods..

[92] Callahan BJ, McMurdie PJ, Rosen MJ, Han AW, Johnson AJ, Holmes SP (2016). DADA2: High-resolution sample inference from Illumina amplicon data. Nat Methods..

[93] Wang Q, Garrity GM, Tiedje JM, Cole JR (2007). Naive Bayesian classifier for rapid assignment of rRNA sequences into the new bacterial taxonomy. Appl Environ Microbiol..

[94] Quast C, Pruesse E, Yilmaz P, Gerken J, Schweer T, Yarza P, Peplies J, Glockner FO (2013). The SILVA ribosomal RNA gene database project: improved data processing and web-based tools. Nucleic Acids Res..

[95] Altschul SF, Gish W, Miller W, Myers EW, Lipman DJ (1990). Basic local alignment search tool. Journal of molecular biology..

[96] Guillou L, Bachar D, Audic S, Bass D, Berney C, Bittner L, Boutte C, Burgaud G, de Vargas C, Decelle J (2013). The protist ribosomal reference database (PR2): a catalog of unicellular eukaryote small sub-unit rRNA sequences with curated taxonomy. Nucleic Acids Res..

[97] Logares R, Sunagawa S, Salazar G, Cornejo-Castillo FM, Ferrera I, Sarmento H, Hingamp P, Ogata H, de Vargas C, Lima-Mendez G (2014). Metagenomic 16S rDNA Illumina tags are a powerful alternative to amplicon sequencing to explore diversity and structure of microbial communities. Environ Microbiol..

[98] Oksanen J, Kindt R, Legendre P, O'Hara B, Simpson GL, Solymos P, Stevens MHH, Wagner H. vegan: Community ecology package. R package version 1.15-0. In*.*; 2008.

[99] Logares R, Audic S, Bass D, Bittner L, Boutte C, Christen R, Claverie JM, Decelle J, Dolan JR, Dunthorn M (2014). Patterns of rare and abundant marine microbial eukaryotes. Curr Biol.

[100] Schloss PD, Westcott SL, Ryabin T, Hall JR, Hartmann M, Hollister EB, Lesniewski RA, Oakley BB, Parks DH, Robinson CJ (2009). Introducing mothur: open-source, platform-independent, community-supported software for describing and comparing microbial communities. Appl Environ Microbiol..

[101] Capella-Gutierrez S, Silla-Martinez JM, Gabaldon T (2009). trimAl: a tool for automated alignment trimming in large-scale phylogenetic analyses. Bioinformatics..

[102] Price MN, Dehal PS, Arkin AP (2009). FastTree: computing large minimum evolution trees with profiles instead of a distance matrix. Mol Biol Evol..

[103] R-Development-Core-Team (2008). R: A language and environment for statistical computing.

[104] Paradis E, Claude J, Strimmer K (2004). APE: Analyses of Phylogenetics and Evolution in R language. Bioinformatics..

[105] Wickham H. ggplot2: Elegant Graphics for Data Analysis: Springer-Verlag; 2009.

[106] Chen J, Bittinger K, Charlson ES, Hoffmann C, Lewis J, Wu GD, Collman RG, Bushman FD, Li H (2012). Associating microbiome composition with environmental covariates using generalized UniFrac distances. Bioinformatics..

[107] Kembel SW, Cowan PD, Helmus MR, Cornwell WK, Morlon H, Ackerly DD, Blomberg SP, Webb CO (2010). Picante: R tools for integrating phylogenies and ecology. Bioinformatics..

[108] Dray S, Blanchet G, Borcard D, Clappe S, Guenard G, Jombart T, Larocque G, Legendre P, Madi N, Wagner HH. adespatial: Multivariate multiscale spatial analysis. In*.*; 2017.

[109] Cavender-Bares J, Kozak KH, Fine PV, Kembel SW (2009). The merging of community ecology and phylogenetic biology. Ecology letters..

[110] Losos JB (2008). Phylogenetic niche conservatism, phylogenetic signal and the relationship between phylogenetic relatedness and ecological similarity among species. Ecol Lett..

[111] Stegen JC, Lin X, Konopka AE, Fredrickson JK (2012). Stochastic and deterministic assembly processes in subsurface microbial communities. ISME J..

[112] Andersson AF, Riemann L, Bertilsson S (2010). Pyrosequencing reveals contrasting seasonal dynamics of taxa within Baltic Sea bacterioplankton communities. ISME J..

[113] Chase JM, Kraft NJB, Smith KG, Vellend M, Inouye BD (2011). Using null models to disentangle variation in community dissimilarity from variation in α-diversity. Ecosphere..

[114] Reshef DN, Reshef YA, Finucane HK, Grossman SR, McVean G, Turnbaugh PJ, Lander ES, Mitzenmacher M, Sabeti PC (2011). Detecting novel associations in large data sets. Science..

[115] Friedman J, Alm EJ (2012). Inferring correlation networks from genomic survey data. PLoS Comput Biol..

[116] Watts SC, Ritchie SC, Inouye M, Holt KE. FastSpar: rapid and scalable correlation estimation for compositional data. Bioinformatics. 2018;35(6):1064-6. 10.1093/bioinformatics/bty734 PMC6419895 30169561

[117] Shannon P, Markiel A, Ozier O, Baliga NS, Wang JT, Ramage D, Amin N, Schwikowski B, Ideker T (2003). Cytoscape: a software environment for integrated models of biomolecular interaction networks. Genome Res..

[118] Csardi G, Nepusz T. The igraph software package for complex network research. InterJournal. 2006; Complex Systems:1695.

