# Supplementary figures and images for: Disentangling the mechanisms shaping the surface ocean microbiota
Source: Microbiome. 2020 Apr 20;8:55. doi: 10.1186/s40168-020-00827-8 (PMC7171866; doi:10.1186/s40168-020-00827-8)

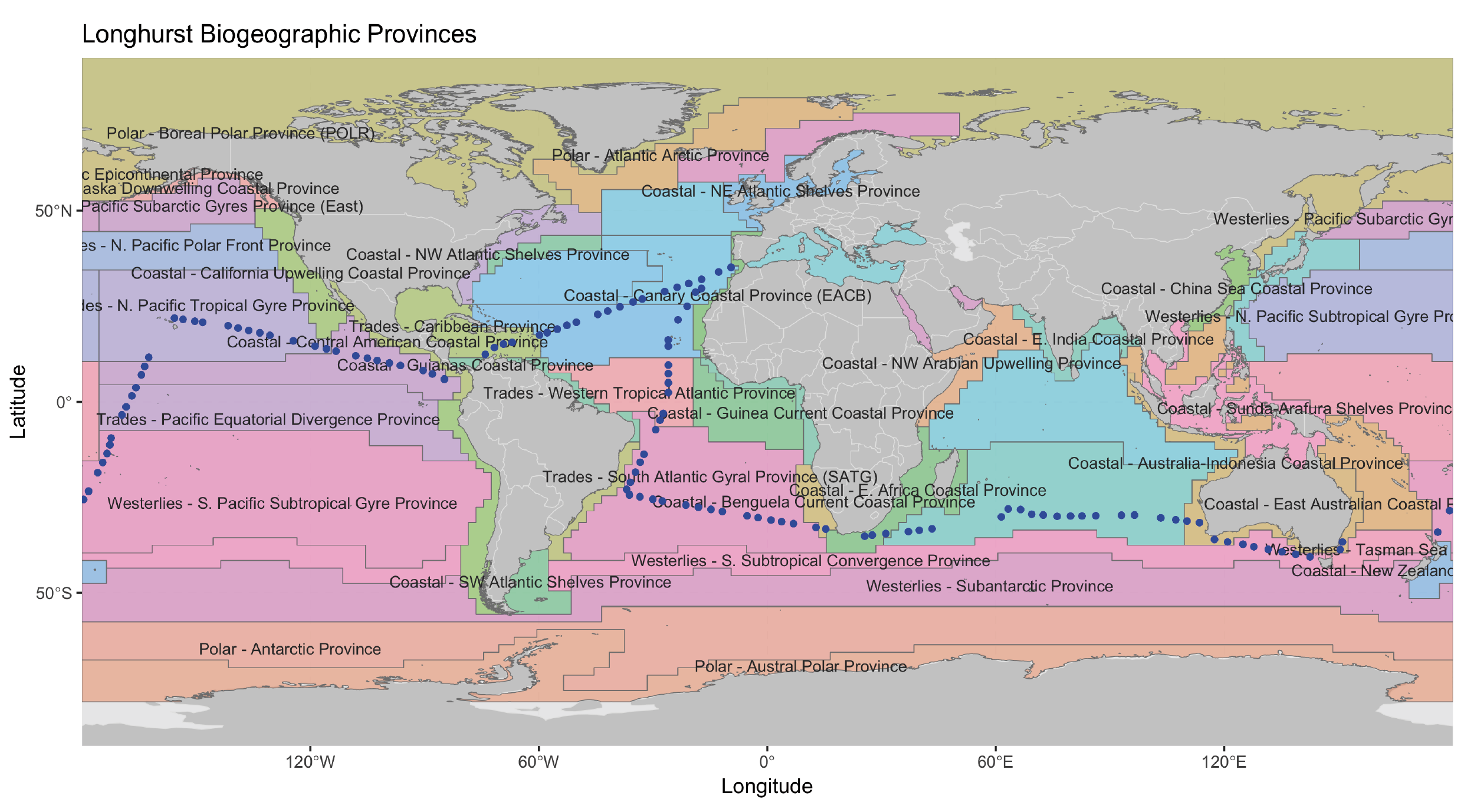

Supplement: Supplementary file 2 — Additional file 1: Figure S1. Position of the 120 analysed Malaspina-2010 stations in the context of the Longhurst biogeographic provinces [37]. [file 40168_2020_827_MOESM1_ESM.png]

**Bray–Curtis**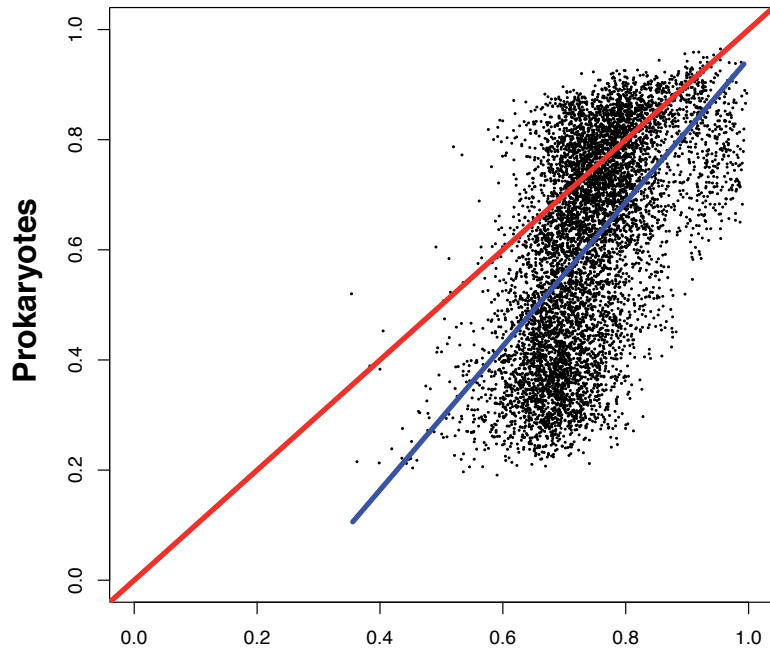**Unifrac**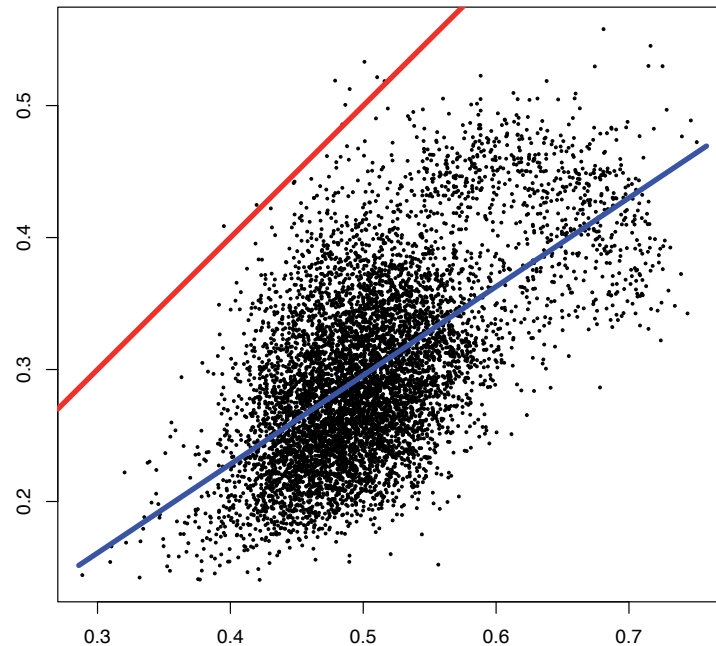**Picoeukaryotes**

Supplement: Supplementary file 3 — Additional file 2: Figure S2. Bray Curtis and gUniFrac distances between picoeukaryotes and prokaryotes from the Malaspina dataset. Regression (blue) and 0:1 (red) lines are indicated. [file 40168_2020_827_MOESM2_ESM.pdf]

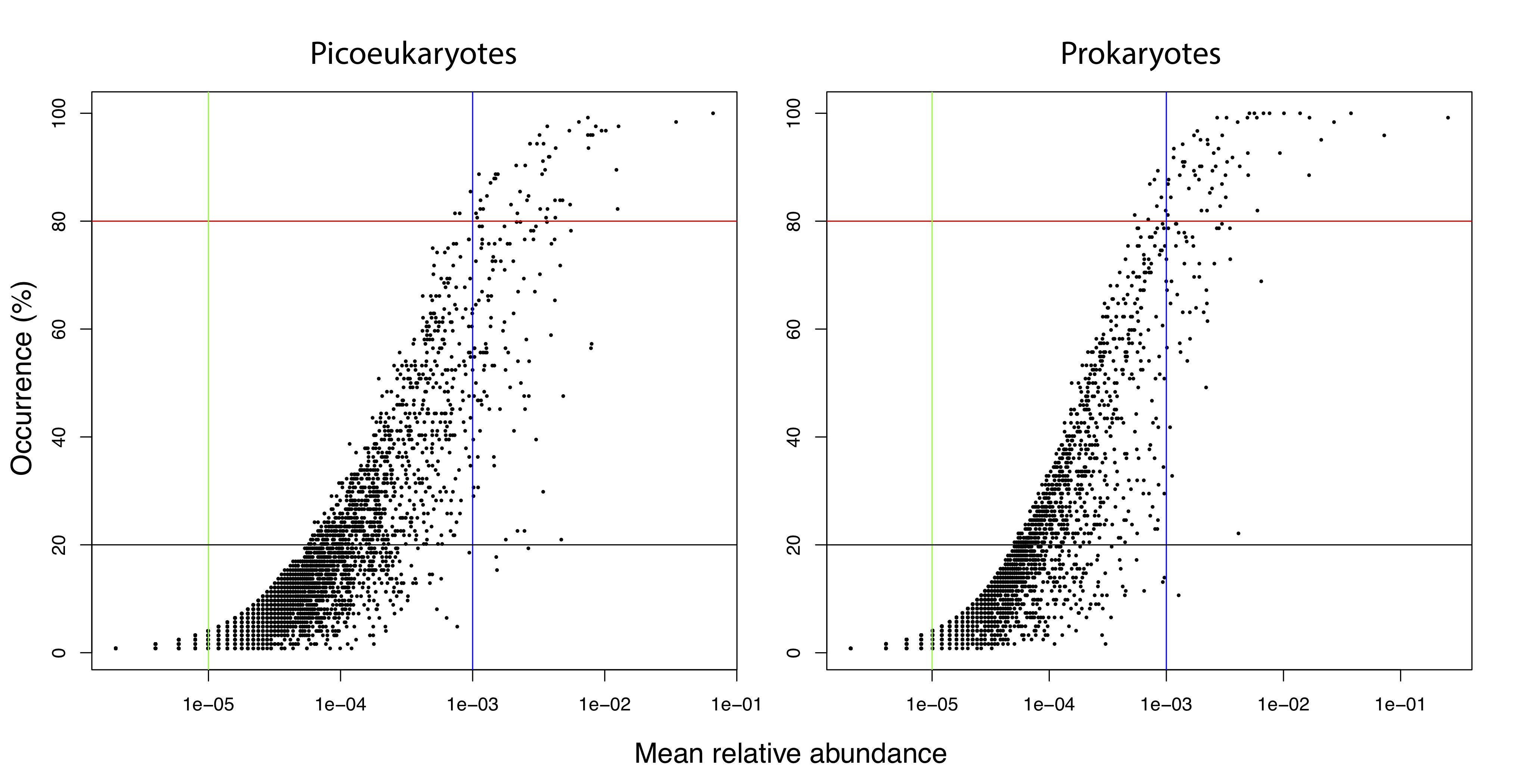

Supplement: Supplementary file 5 — Additional file 4: Figure S3. OTUs-99% mean relative abundance (i.e. regional abundance) vs. occurrence (i.e. number of samples in which each OTUs-99% is present) for the Malaspina dataset. The red and black horizontal lines indicate percentages of occurrences of 80% and 20% respectively. Cosmopolitan OTUs were considered as those with a percentage of occurrence >80%, while restricted OTUs were those with a percentage of occurrence <20% (see Table S2, Additional file 5). Blue and green vertical lines indicate regional abundances above and below which OTUs are considered regionally abundant (>0.1%) or rare (<0.001%) respectively. [file 40168_2020_827_MOESM4_ESM.png]

Latitude

Longitude

50

0

-50

-100

0

100

200

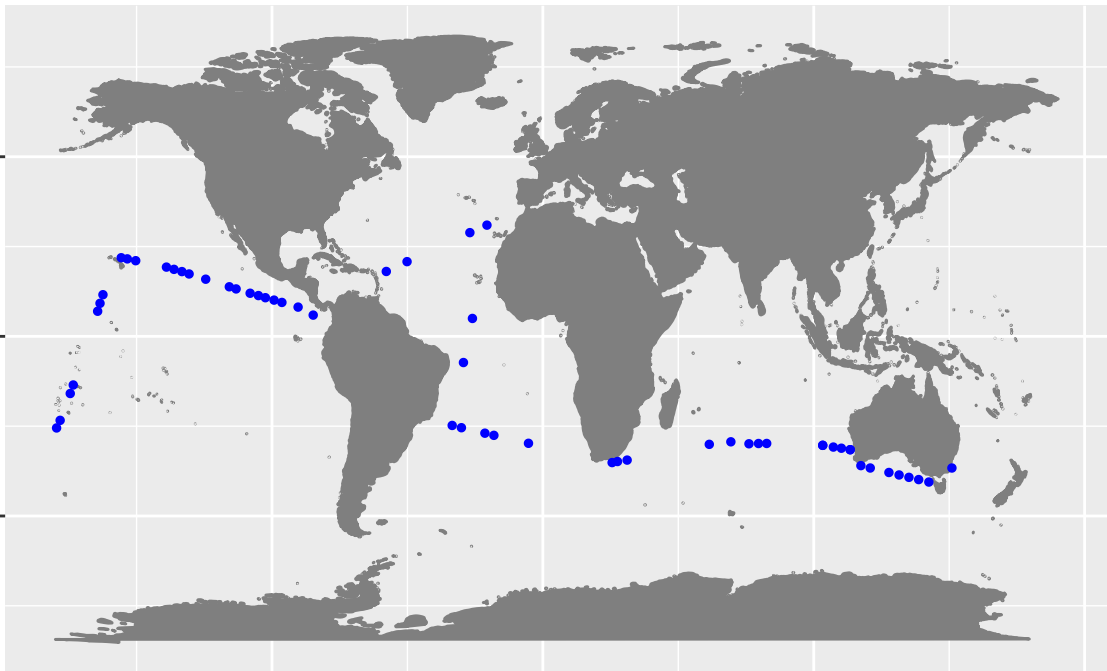

Supplement: Supplementary file 8 — Additional file 7: Figure S4. The 57 Malaspina stations for which 17 environmental parameters were available (Meta-57 dataset). [file 40168_2020_827_MOESM7_ESM.pdf]

# Water temperature

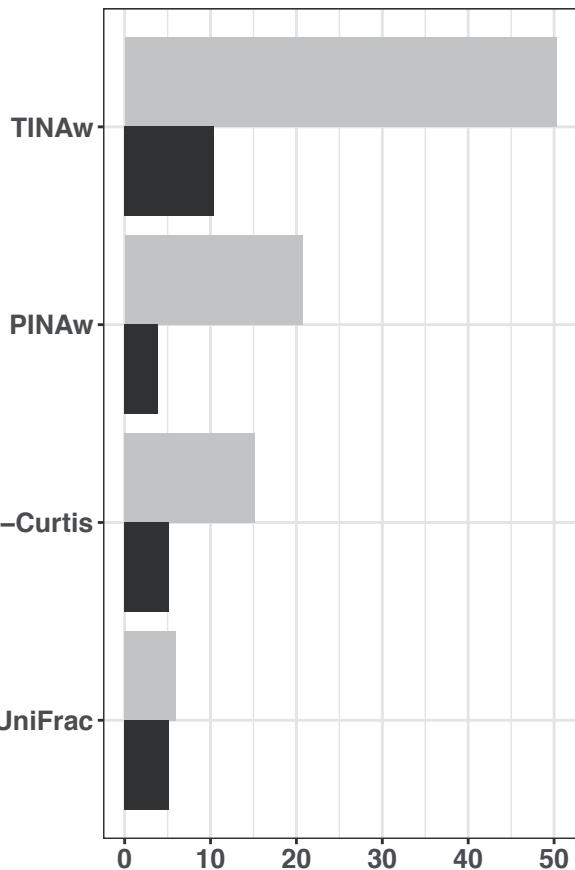

# Fluorescence

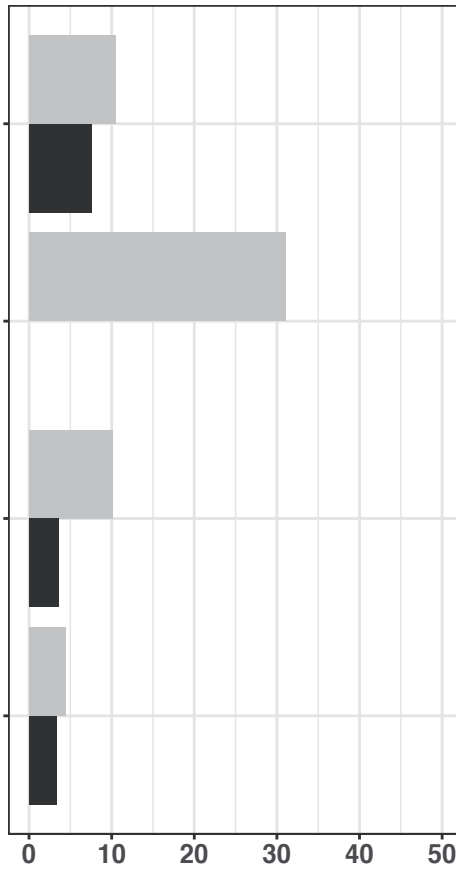

Prokaryotes  
Picoeukaryotes

Percentage (ADONIS R<sup>2</sup>)

Supplement: Supplementary file 9 — Additional file 8: Figure S5. Percentage of variance in Picoeukaryotic and Prokaryotic community composition (ADONIS R2) explained by water temperature and fluorescence when using different β-diversity metrics. Figure based on the Malaspina Meta-57 dataset. [file 40168_2020_827_MOESM8_ESM.pdf]

## OTUs

## Abundance

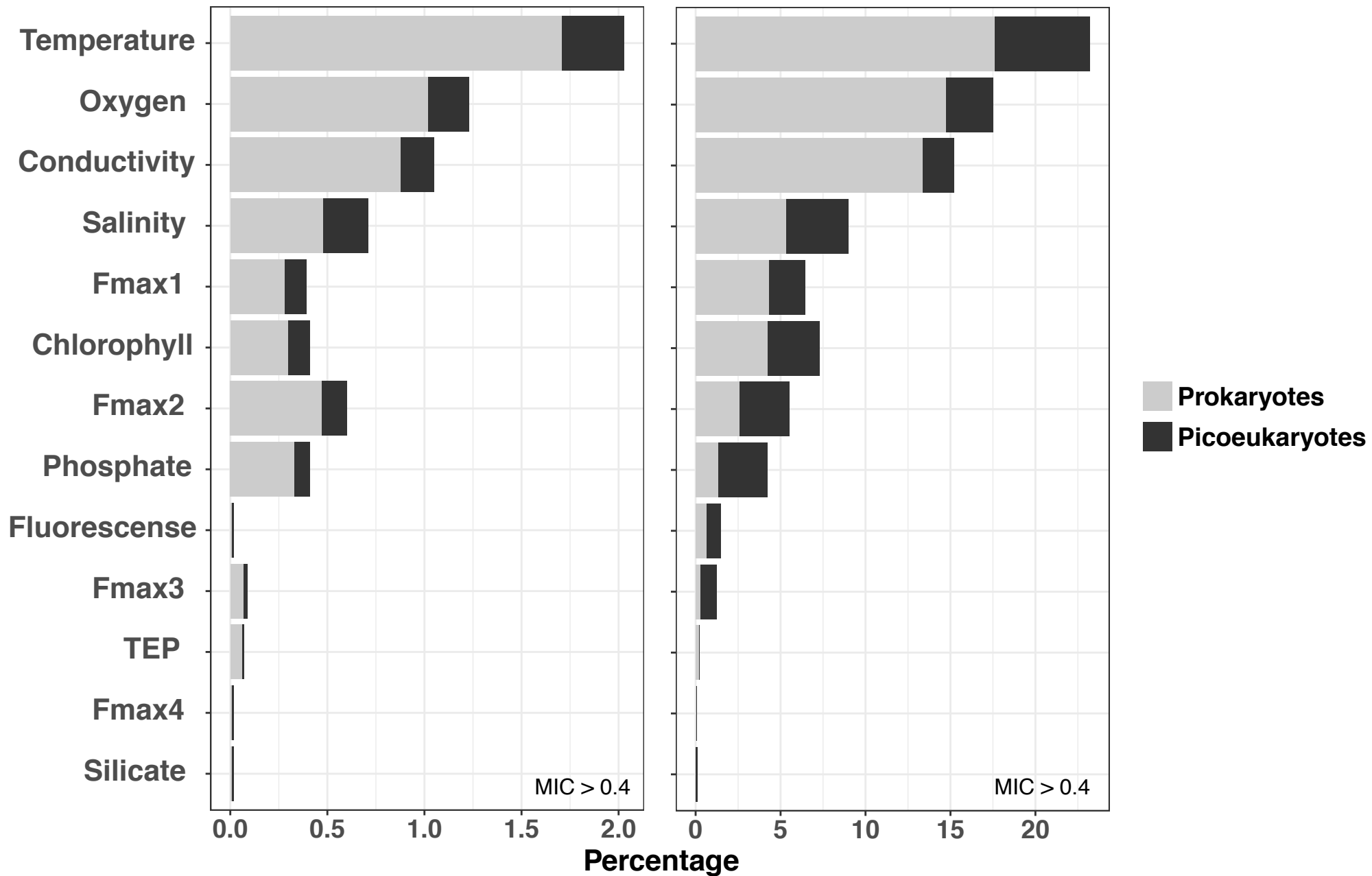

Supplement: Supplementary file 14 — Additional file 13: Figure S7. Percentage of OTUs-99% significantly associated to different environmental variables (MIC > 0.4) [left] and their corresponding contribution to total sequence abundance (i.e. percentage of reads) [right] in the Malaspina dataset. NB: Temperature, Oxygen, Conductivity and Salinity are correlated. OTUs can be associated to more than one variable. [file 40168_2020_827_MOESM13_ESM.pdf]

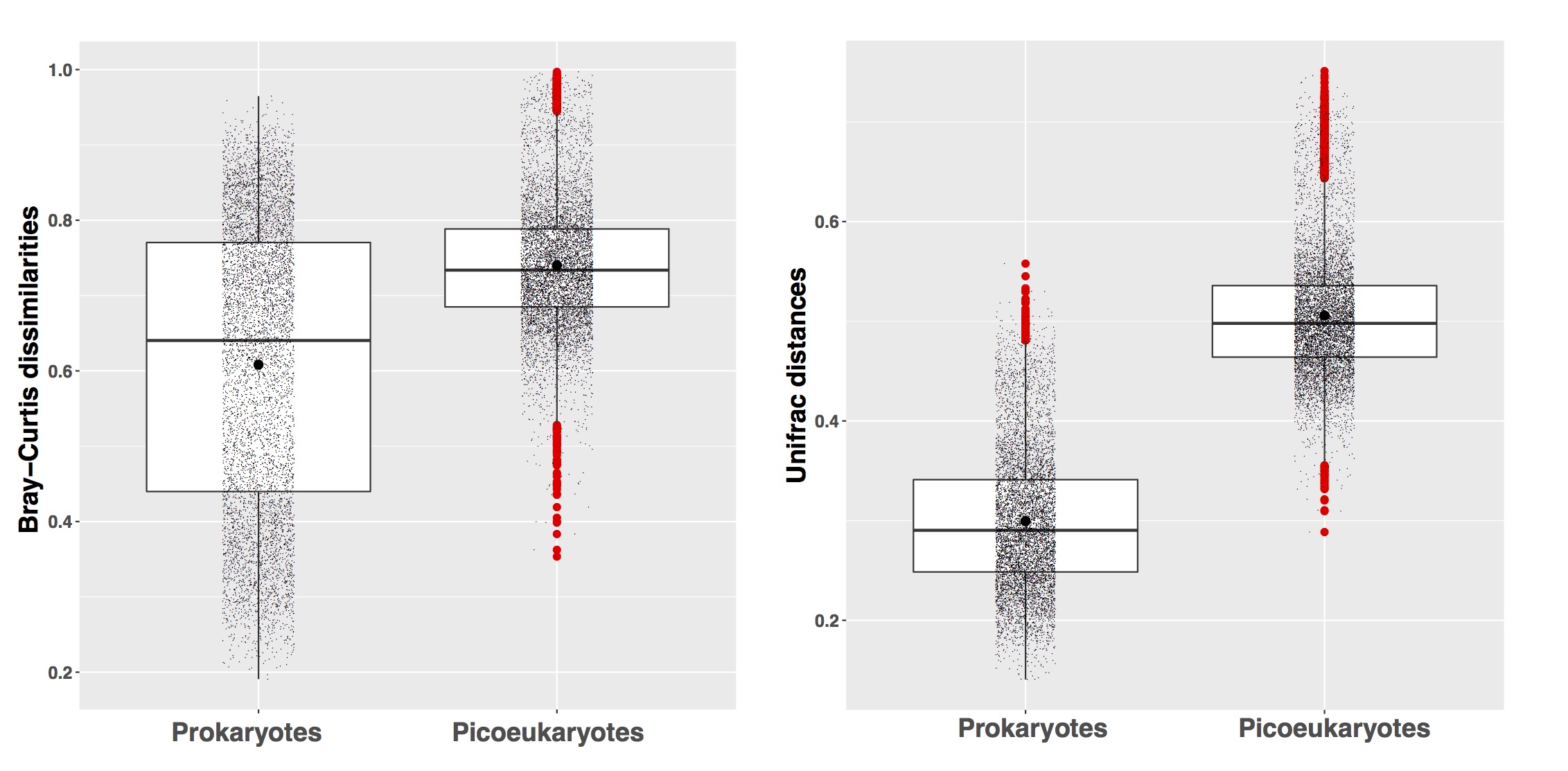

Supplement: Supplementary file 16 — Additional file 15: Figure S8. Bray-Curtis dissimilarities and gUniFrac distances in Prokaryotes and Picoeukaryotes from the Malaspina dataset. In both cases, mean differences were significant (Wilcoxon text, p<0.05). Prokaryotes (Bray Curtis mean=0.61, SD=0.19; gUniFrac mean=0.30, SD=0.07); Picoeukaryotes (Bray Curtis mean=0.74, SD=0.08; gUniFrac mean=0.50, SD=0.06). [file 40168_2020_827_MOESM15_ESM.jpg]

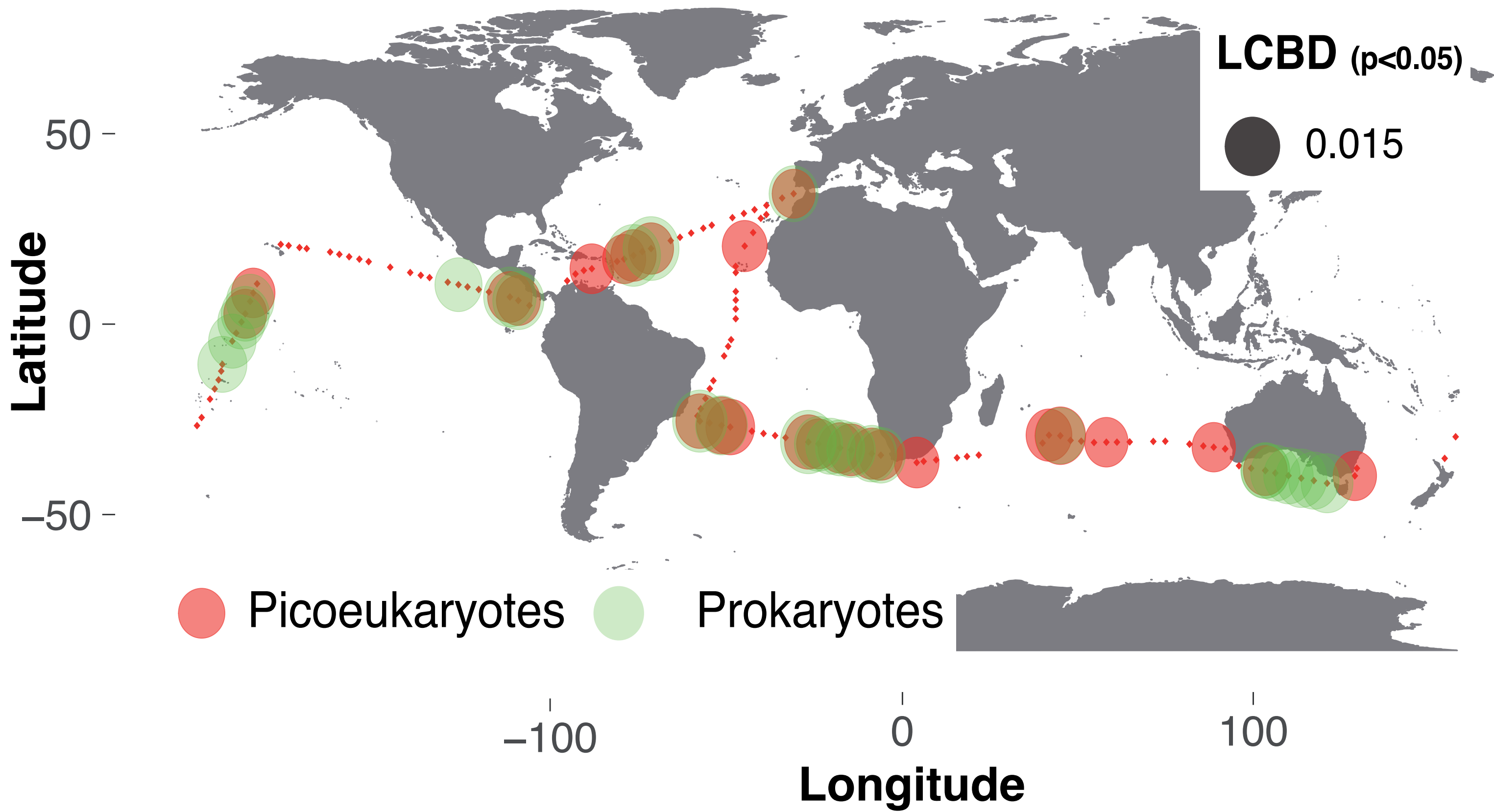

Supplement: Supplementary file 17 — Additional file 16: Figure S9. Stations (total 36) from the Malaspina dataset featuring a comparatively large contribution to the overall β-diversity (LCBD = Local Contributions to Beta Diversity [38]; p<0.05). [file 40168_2020_827_MOESM16_ESM.pdf]
