# Supplementary material for: Disentangling the mechanisms shaping the surface ocean microbiota
Source: Microbiome. 2020 Apr 20;8:55. doi: 10.1186/s40168-020-00827-8 (PMC7171866; doi:10.1186/s40168-020-00827-8)
Supplement: Supplementary file 4 — Additional file 3: Table S1. Regionally abundant or rare prokaryotic and picoeukaryotic OTUs-99% from the Malaspina dataset. [file 40168_2020_827_MOESM3_ESM.docx]

**Table S1.** Regionally abundant or rare prokaryotic and picoeukaryotic OTUs_-99%_

from the *Malaspina* dataset.

|  | **Prokaryotes (%)** | **Picoeukaryotes (%)** |
| --- | --- | --- |
| Regionally abundant OTUs (>0.1%)^1^ | 1.46 (103) | 0.84 (158) |
| Regionally rare OTUs (<0.001%)^2^ | 47.6 (3,343) | 71.5 (13,499) |

^1^ OTUs featuring a mean relative abundance >0.1%. ^2^ OTUs featuring a mean relative abundance <0.001%. Percentages as well as corresponding number of OTUs (within parenthesis) are indicated.
