# Supplementary material for: Disentangling the mechanisms shaping the surface ocean microbiota
Source: Microbiome. 2020 Apr 20;8:55. doi: 10.1186/s40168-020-00827-8 (PMC7171866; doi:10.1186/s40168-020-00827-8)
Supplement: Supplementary file 6 — Additional file 5: Table S2. OTUs-99% displaying Cosmopolitan, Intermediate and Restricted distributions in the Malaspina dataset. [file 40168_2020_827_MOESM5_ESM.docx]

Table S2. OTUs_-99%_ displaying Cosmopolitan, Intermediate and Restricted distributions in the *Malaspina* dataset.

|  | **Cosmopolitan (>80%)^1^** | **Intermediate (80-20%)^2^** | **Restricted (<20%)^3^** |
| --- | --- | --- | --- |
| **Picoeukaryotes** | 0.3 (57) | 5.1 (954) | 94.6 (17,870) |
| **Prokaryotes** | 1.0 (68) | 11.1 (779) | 87.9 (6,178) |

^1^ Percentage of OTUs occurring in >80% of the samples (Cosmopolitan). ^2^ Percentage of OTUs occurring in 80-20% of the samples (Intermediate distribution). ^3^ Percentage of OTUs occurring in <20% of the samples (Restricted distribution). The number of OTUs is indicated within parentheses.
