# Supplementary material for: Disentangling the mechanisms shaping the surface ocean microbiota
Source: Microbiome. 2020 Apr 20;8:55. doi: 10.1186/s40168-020-00827-8 (PMC7171866; doi:10.1186/s40168-020-00827-8)
Supplement: Supplementary file 7 — Additional file 6: Supplementary Methods. [file 40168_2020_827_MOESM6_ESM.pdf]

SUPPLEMENTARY METHODS

Sample collection

Surface waters (3 m depth) from a total of 120 globally-distributed stations located in the tropical and sub-tropical global ocean **(Figure 1A)** were sampled from December 2010 to July 2011 as a part of the *MALASPINA-2010* expedition [1] conducted on the R/V Hespérides. Water samples were obtained with a large (20 L) Niskin bottle deployed simultaneously to a CTD profiler that included sensors for conductivity, temperature, oxygen, fluorescence and turbidity. After collection, ≈12 L of seawater were subsequently pre-filtered through a 200 µm nylon mesh to remove large plankton, and then sequentially filtered, using a peristaltic pump, through a 20 µm nylon mesh (at the entrance of the tubing) and 3 µm and 0.2 µm polycarbonate filters of 47 mm diameter (Isopore, Millipore, Burlington, MA, USA). Filtration time was ≈15 minutes. After filtration, filters were flash-frozen in liquid N_2 and stored at -80 ºC until downstream analyses. Samples for inorganic nutrients (NO_3^-, NO_2^-, PO_4^{3-}, SiO_2) were collected from the Niskin bottle, kept frozen, and measured spectrophotometrically using an Alliance Evolution II autoanalyzer (Frépillon, France) [2]. In specific samples, where the previous method failed or was not applied, we estimated nutrient concentration using the World Ocean Database [3]. Given that not all environmental parameters were available for all stations, two contextual datasets were generated: *Meta-119*, including 119 stations, 5 environmental parameters and 5 spatial features **(Figure 1A)** and *Meta-57*, considering 57 stations and 17 environmental parameters (See below; **Figure S4, Additional file 7**). In statistical analyses, continuous environmental variables were standardized as z-scores, that is, deviations of the values from the global mean in standard deviation units.

DNA extraction, amplicon sequencing and bioinformatic analyses

DNA was extracted using a standard phenol-chloroform protocol [4]. Fragments from both the 18S and 16S rRNA-genes were amplified from the same DNA extracts. The hypervariable V4 region of the 18S rRNA genes (≈380 bp) was amplified with the primers TAReukFWD1 and TAReukREV3 [5], while the hypervariable V4-V5 (≈400bp) region of the 16S rRNA genes was amplified with the primers 515F-Y - 926R [6], which target both Bacteria and Archaea. Samples were amplified for sequencing in a two-step process. In the first step, the forward primer was constructed with the Illumina i5 sequencing primer (5'-TCGTCGGCAGCGTCAGATGTGTATAAGAGACAG-3') and the TAReukFWD1 (18S rRNA genes) or 515F-Y (16S rRNA genes) primers. The reverse primer was constructed with the Illumina i7 sequencing primer (5'-GTCTCGTGGGCTCGGAGATGTGTATAAGAGACAG-3') and the TAReukREV3 (18S rRNA genes) or 926R (16S rRNA genes) primers. Amplifications were performed in 25 μl reactions with Qiagen HotStar Taq master mix (Qiagen Inc., Valencia, CA, USA), 1μl of each 5 μM primer, and 1 μl of template. Reactions were performed on ABI Veriti thermocyclers (Applied Biosystems, Carlsbad, CA, USA) under the following thermal "touchdown" profile: 95 °C for 5 min, then 10 cycles of 94 °C for 30 sec, 50 °C for 40 sec (+0.5 °C per cycle), 72 °C for 1 min, followed by 25 cycles of 94 °C for 30 sec, 54 °C for 40 sec, 72 °C for 1 min, and finally, one cycle of 72 °C for 10 min. Products from the first amplification step were added to a second PCR based on qualitatively determined concentrations. Primers for the second PCR were designed based on the Illumina Nextera PCR primers as follows: Forward -5'-AATGATACGGCGACCACCGAGATCTACAC[i5index]TCGTCGGCAGCGTC-3' and Reverse -5'-CAAGCAGAAGACGGCATACGAGAT[i7index]GTCTCGTGGGCTCGG-3'. The

second stage amplification consisted in 95 °C for 5 min, then 10 cycles of 94 °C for 30 sec, 54 °C for 40 sec, 72 °C for 1 min, followed by one cycle of 72 °C for 10 min.

Amplification products were visualized with eGels (Life Technologies, Grand Island, NY, USA). Products were then pooled equimolar and each pool was size selected in two rounds using Agencourt AMPure XP (BeckmanCoulter, Indianapolis, IN, USA) in a 0.75 ratio for both rounds. Size selected pools were then quantified using the Qubit 2.0 fluorometer (Life Technologies) and loaded on an *Illumina* MiSeq (Illumina, Inc. San Diego, CA, USA) flow cell at 10 pM. Sequencing was performed using 2x250 bp. Amplicon library construction and sequencing was performed at the Research and Testing Laboratory facility (Lubbock, TX, USA; http://www.researchandtesting.com/).

A total of 71,391,060 (2 x 35,695,530) reads were produced for picoeukaryotes, while 17,129,672 (2 x 8,564,836) reads were produced for prokaryotes. Reads were processed following an in-house pipeline [7]. Briefly, raw reads were corrected using BayesHammer [8] following Schirmer et al. [9] Corrected paired-end reads were subsequently merged with PEAR [10] and sequences longer than 200 bp were quality-checked (maximum expected errors [maxEE] = 0.5) and de-replicated using USEARCH V8.1.1756 [11]. OTUs were delineated at 99% similarity using UPARSE V8.1.1756 [12]. To obtain OTU abundances, reads were mapped back to OTUs at 99% similarity using an exhaustive search *(-maxaccepts 20 -maxrejects 50,000-100,000*). Chimera check and removal was performed both *de novo* and using the SILVA reference database [13]. After our stringent quality control, a total of 16,460,248 18S rRNA and 5,697,779 16S rRNA gene-sequences were left, which were associated to 42,505 18S and 10,158 16S OTUs. See more details on sequence processing in **Table S8, Additional file 18**. Taxonomic assignment of 18S OTUs was generated by BLASTing [14] OTU-representative sequences against three reference databases, PR^2 [15] and two in-house marine protist

databases (available at https://github.com/ramalok), one based on a collection of Sanger sequences from molecular surveys [16] and the other based on 454 reads from the BioMarKs project [17]. Metazoan, Streptophyta and nucleomorph OTUs were removed. Similarly, for 16S rRNA OTUs, taxonomic assignment was based on BLASTing OTU-representative sequences against SILVA v123. Chloroplast and mitochondrial sequences were removed. BLAST hits were filtered prior to taxonomy assignment using an in-house python script, considering a percentage of identity >90%, a coverage >70%, a minimum alignment length of 200 bp and an e-value < 0.00001.

Additionally, to investigate the effects of clustering on the estimation of ecological mechanisms, we determined OTUs as Amplicon Sequence Variants (ASVs) using DADA2 [18]. For the 18S, we trimmed the forward reads at 240 bp and the reverse reads at 180 bp, while for the 16S, forward reads were trimmed at 220 bp and reverse reads at 200 bp. Then, for the 18S, the maximum number of expected errors (maxEE) was set to 7 and 8 for the forward and reverse reads respectively, while for the 16S, the maxEE was set to 2 for the forward reads and to 4 for the reverse reads. Error rates for each possible nucleotide substitution type were estimated using a machine learning approach implemented in DADA2 for both the 18S and 16S. Error rate estimations of the 18S were based on ≈284 million and ≈213 million bases for the forward and reverse reads respectively, while for the 16S ≈224 million and ≈204 million bases were used for the forward and reverse reads respectively. When considering quality scores between 0 – 40, error rates of the 18S ranged between ≈0.0002 and 0.25 in the forward reads and between $\approx 8.6 \times 10^{-5}$ and 0.25 in the reverse reads. In turn, for the 16S, error rates for the forward reads ranged between ≈0.0004 and 0.25, while for the reverse reads they ranged between ≈0.0007 and 0.25. As expected, error rates decreased with increasing quality score. Estimated error rates were used in DADA2 to delineate unique variants or $OTUs_{\text{-ASVs}}$.

A total of 21,970 and 6,196 $OTUs_{-ASVs}$ were delineated for the 18S and 16S respectively. $OTUs_{-ASVs}$ were assigned taxonomy using the naïve Bayesian classifier method [19] together with the SILVA version 132 [13] database as implemented in DADA2. Eukaryotic ASVs were also BLASTed [14] against the Protist Ribosomal Reference database (PR^2, version 4.11.1; [15]). Streptophyta, Metazoa, nucleomorphs, chloroplasts and mitochondria were removed from $OTUs_{-ASVs}$ tables. Tables of $OTUs_{-ASVs}$ were rarefied to 20,000 reads per sample with the function *rrarefy* in Vegan. Only $OTUs_{-ASVs}$ with abundances >100 reads were used for the calculation of ecological mechanisms **(Figure 1B)**.

Computing analyses were performed at the MARBITS bioinformatics platform of the Institut de Ciències del Mar (ICM; http://marbits.icm.csic.es) as well as in MareNostrum (Barcelona Supercomputing Center). Sequences are publicly available at the European Nucleotide Archive (http://www.ebi.ac.uk/ena; accession numbers PRJEB23913 [18S rRNA genes] & PRJEB25224 [16S rRNA genes])

Statistical analyses and phylogenetic inferences

In order to allow for comparisons, both picoeukaryotic and prokaryotic $OTUs_{-99\%}$ datasets were sub-sampled to 4,060 reads per sample using *rrarefy* in *Vegan* [20]. The sub-sampled picoeukaryotic and prokaryotic OTU tables contained 18,775 and 7,025 OTUs respectively. All OTUs with mean relative abundances above 0.1% and below 0.001% were defined as regionally abundant or rare respectively [21].

Most analyses and graphs were performed in the R statistical environment [22] using *ggplot2* [23], *Maps* [24], *Mapplots* [25] and *Vegan*. Local Contributions to Beta Diversity [26] was measured with *adespatial* [27]. Phylogenetic trees were constructed for both the 16S and 18S rRNA gene-datasets using $OTUs_{-99\%}$ representative sequences

or $OTUs_{\text{-ASVs}}$. Reads were aligned against an aligned SILVA template using mothur [28]. Afterwards, poorly aligned regions or sequences were removed using trimAl (parameters: -gt 0.3 -st 0.001) [29]. Phylogenetic trees were inferred with FastTree [30] using the Generalized Time Reversible (GTR) model of nucleotide substitution considering a CAT/Gamma-distributed rate of variation across sites (including 20 rate categories). Other phylogenetic analyses were performed with the R-packages *picante* [31], *APE* [32] and *gUniFrac* [33]. *gUniFrac* was run with an alpha value of 0.5.

Quantification of Selection, Drift and Dispersal

These processes were quantified using the approach proposed by Stegen et al. [34]. This methodology consists of two main sequential steps: the first uses OTU phylogenetic turnover to infer the action of selection and the second uses OTU compositional turnover to infer the action of dispersal and drift. The action of selection, dispersal and drift was quantified using both $OTUs_{\text{-99\%}}$ and $OTUs_{\text{-ASVs}}$. Before using phylogenetic turnover, we tested for phylogenetic signal using temperature and fluorescence, which were the two variables that explained the highest fraction of community variance. We found phylogenetic signal across relatively short phylogenetic distances **(Figure S10, Additional file 19**; **Figure S11, Additional file 20)** for temperature and fluorescence. Other unmeasured environmental variables could also have phylogenetic signal for the 16S and 18S. In such cases, the implemented approach [34] would detect that phylogenetic pattern and associate it to *selection*, even though the environmental variable that generated it was not measured.

Short phylogenetic distances indicate that phylogenetic turnover should be measured among close relatives [35]. For this reason, phylogenetic turnover was measured using the abundance-weighted β-Mean Nearest Taxon Distance (βMNTD)

metric [34, 36], which quantifies the mean phylogenetic distances between the evolutionary closest OTUs in two communities. βMNTD values can be larger, smaller or equal to the values expected when selection is not affecting community turnover (that is, expected under a random distribution). βMNTD values higher than expected indicate that communities are under heterogeneous selection [36]. In contrast, βMNTD values which are lower than expected indicate that communities are experiencing homogeneous selection. Null models were constructed using 999 randomizations as in Stegen et al. [34]. Differences between the observed βMNTD values and the mean of the null distribution are denoted as β-Nearest Taxon Index (βNTI), with | βNTI | > 2 being considered as significant departures from random phylogenetic turnover, pointing to the action of selection.

The second step of this process calculates whether the observed β-diversity, based in OTU turnover, could be generated by drift or other processes. For this, we calculated the Raup-Crick metric [37] using Bray-Curtis dissimilarities (hereafter RC_{bray}), following Stegen et al. [34]. RC_{bray} compares the measured β-diversity against the β-diversity that would be obtained if drift was driving community turnover (that is, under random community assembly). Randomizations were run 9,999 times and only OTUs with >100 reads over the entire dataset were considered. RC_{bray} values between -0.95 and +0.95 point to a community assembly governed by drift. On the contrary, RC_{bray} values > +0.95 or < -0.95 indicate that community turnover is driven by low/high dispersal respectively [37]. According to Stegen et al. [34], dispersal limitation is only expected to produce significant RC_{bray} values when coupled to drift, which introduces stochastic changes in community composition that magnify their differentiation leading eventually to RC_{bray} values > +0.95. In contrast, homogenizing dispersal (similar to mass effects) could

generate RC_{bray} values < -0.95, reflecting a process in which the composition of two communities is more similar than expected by chance due to high immigration rates.

The previous framework was applied as proposed by Stegen et al. [34]: First, we determined the fraction of total pairwise comparisons with a |βNTI| > 2. This proportion was interpreted as the overall action of selection in our global surface-ocean picoplankton dataset. As a consequence, the turnover of communities featuring |βNTI| < 2 should be driven by dispersal limitation, homogenizing dispersal or drift. Thus, the second step in this procedure was to calculate the RC_{bray} for all those community pairs whose turnover was not governed by selection (that is, those with |βNTI| < 2). Here, values of RC_{bray} > +0.95 are interpreted as dispersal limitation, values of RC_{bray} < -0.95 are interpreted as homogenizing dispersal, while values of | RC_{bray} | < +0.95 are associated to drift. Thus, for the pairwise comparisons that did not indicate the action of selection, we calculated the proportion of total comparisons that could be assigned to dispersal limitation, homogenizing dispersal or drift according to their RC_{bray} values.

Environmental datasets

The *Meta-119* dataset, included 119 stations, 5 environmental parameters, and 5 spatial features for most stations. The 5 environmental parameters were: Temperature (ºC), Conductivity (S m^{-1}), Fluorescence, Salinity and Dissolved Oxygen (ml L^{-1}). *Meta-119* also considered the following spatial features: Longhurst Provinces [38] (**Figure S1, Additional file 1**), Ocean (Atlantic, Indian, Pacific), Ocean Subdivision (Indian, North Atlantic, North Pacific, Pacific, South Atlantic, South Australian Bight, South Pacific), Distance to the coast <370 km and Terrestrial influence.

The *Meta-57* dataset considered 57 stations **(Figure S4, Additional file 7)** and 17 environmental parameters for most stations. The 17 environmental parameters were:

Temperature (ºC), Conductivity (S m^{-1}), Fluorescence, PAR (Photosynthetically Active Radiation; measured with a sensor attached to the CTD), Turbidity, Salinity, Dissolved Oxygen (ml L^{-1}), Chlorophyll concentration (µg L^{-1}) [39], Fluorescent Dissolved Organic Matter (FDOM; four peaks associated to humic and amino-acid substances were measured, indicated as Fmax1, Fmax2, Fmax3, Fmax4; see [40]), TEP (Transparent Exopolymer Particles) [41], POC (Particulate Organic Carbon) [41], NO_3_Mala_WOA13 (µmol L^{-1}) [Nitrate, values from *Malaspina* and WOA13], PO_4_Mala_WOA13 (µmol L^{-1}) [Phosphate, values from *Malaspina* and WOA13], SiO_4_Mala_WOA13 (µmol L^{-1}) [Silicate, values from *Malaspina* and WOA13] [40, 42, 43].

Maximal Information Coefficient (MIC) analyses

In MIC analyses [44], the 17 environmental parameters used in the *Meta-57* dataset were considered (see above ***Environmental datasets***). In analyses of picoeukaryotic or prokaryotic OTUs vs. environmental parameters, all $OTUs_{-99\%}$ were considered, while in analyses including comparisons of all $OTUs_{-99\%}$ against each other plus environmental parameters, only $OTUs_{-99\%}$ with ≥100 reads were included, due to computational limitations.

MIC analyses using the *TARA Oceans* datasets included 8 environmental parameters for prokaryotes (63 stations): Temperature (ºC), Salinity, Oxygen (µmol/kg), NO_3 (µmol/L), NO_2 (µmol/L), PO_4 (µmol/L), NO_2NO_3 (µmol/L) and SI (µmol/L). These data are publicly available in: http://ocean-microbiome.embl.de/companion.html. MIC analyses of microbial eukaryotes from *TARA Oceans* considered 6 environmental parameters (40 stations / 41 samples): Temperature (ºC), Salinity, Oxygen (µmol/kg), NO_3 (µmol/L), PAR, Chlorophyll *a* (mg/m^3). These data are available in: http://taraoceans.sb-roscoff.fr/EukDiv/. In MIC analyses of OTUs against environmental

parameters, only OTUs with ≥ 30 reads were used for both microbial eukaryotes (10,115 OTUs, 61,407,151 reads) and prokaryotes (5,029 OTUs, 6,402,539 reads). Given the large number of possible pairwise comparisons in analyses considering all OTUs and environmental parameters against each other, only OTUs with ≥ 500 reads were used for prokaryotes (1,656 OTUs, 5,930,665 reads) while OTUs with ≥ 1,000 reads were used for microbial eukaryotes (2,026 OTUs, 59,897,456 reads). Non-linear associations were defined as MIC-ρ^2 >0.2 [44].

REFERENCES

1. Duarte CM. Seafaring in the 21St Century: The Malaspina 2010 Circumnavigation Expedition. Limnology and Oceanography Bulletin. 2015; 24(1):11-14.
2. Grasshoff K, Ehrhardt M, Kremling K. Methods on seawater analysis; 1983.
3. Boyer TP, Antonov JI, Baranova OK, Coleman C, Garcia HE, Grodsky A, Johnson DR, Locarnini RA, Mishonov AV, O'Brien TD *et al*. World Ocean Database 2013. In: *NOAA Atlas NESDIS 72.* Edited by Levitus S, Mishonov A. Silver Spring, MD: NOAA; 2013.
4. Massana R, Murray AE, Preston CM, DeLong EF. Vertical distribution and phylogenetic characterization of marine planktonic Archaea in the Santa Barbara Channel. Appl Environ Microbiol. 1997; 63(1):50-56.
5. Stoeck T, Bass D, Nebel M, Christen R, Jones MD, Breiner HW, Richards TA. Multiple marker parallel tag environmental DNA sequencing reveals a highly complex eukaryotic community in marine anoxic water. Mol Ecol. 2010; 19 Suppl 1:21-31.
6. Parada AE, Needham DM, Fuhrman JA. Every base matters: assessing small subunit rRNA primers for marine microbiomes with mock communities, time series and global field samples. Environ Microbiol. 2016; 18(5):1403-1414.
7. Logares R. Workflow for Analysing MiSeq Amplicons based on Uparse v1.5. In.: https://doi.org/10.5281/zenodo.259579; 2017.
8. Nikolenko SI, Korobeynikov AI, Alekseyev MA. BayesHammer: Bayesian clustering for error correction in single-cell sequencing. BMC Genomics. 2013; 14 Suppl 1:S7.
9. Schirmer M, Ijaz UZ, D'Amore R, Hall N, Sloan WT, Quince C. Insight into biases and sequencing errors for amplicon sequencing with the Illumina MiSeq platform. Nucleic Acids Res. 2015; 43(6):e37.
10. Zhang J, Kobert K, Flouri T, Stamatakis A. PEAR: a fast and accurate Illumina Paired-End reAd mergeR. Bioinformatics. 2014; 30(5):614-620.
11. Edgar RC. Search and clustering orders of magnitude faster than BLAST. Bioinformatics. 2010; 26(19):2460-2461.
12. Edgar RC. UPARSE: highly accurate OTU sequences from microbial amplicon reads. Nat Methods. 2013; 10(10):996-998.

13. Quast C, Pruesse E, Yilmaz P, Gerken J, Schweer T, Yarza P, Peplies J, Glockner FO. The SILVA ribosomal RNA gene database project: improved data processing and web-based tools. Nucleic Acids Res. 2013; 41(Database issue):D590-596.
14. Altschul SF, Gish W, Miller W, Myers EW, Lipman DJ. Basic local alignment search tool. Journal of molecular biology. 1990; 215(3):403-410.
15. Guillou L, Bachar D, Audic S, Bass D, Berney C, Bittner L, Boutte C, Burgaud G, de Vargas C, Decelle J *et al*. The Protist Ribosomal Reference database (PR2): a catalog of unicellular eukaryote small sub-unit rRNA sequences with curated taxonomy. Nucleic Acids Res. 2013; 41(Database issue):D597-604.
16. Pernice MC, Logares R, Guillou L, Massana R. General patterns of diversity in major marine microeukaryote lineages. PLoS One. 2013; 8(2):e57170.
17. Massana R, Gobet A, Audic S, Bass D, Bittner L, Boutte C, Chambouvet A, Christen R, Claverie JM, Decelle J *et al*. Marine protist diversity in European coastal waters and sediments as revealed by high-throughput sequencing. Environ Microbiol. 2015; 17(10):4035-4049.
18. Callahan BJ, McMurdie PJ, Rosen MJ, Han AW, Johnson AJ, Holmes SP. DADA2: High-resolution sample inference from Illumina amplicon data. Nat Methods. 2016; 13(7):581-583.
19. Wang Q, Garrity GM, Tiedje JM, Cole JR. Naive Bayesian classifier for rapid assignment of rRNA sequences into the new bacterial taxonomy. Appl Environ Microbiol. 2007; 73(16):5261-5267.
20. Oksanen J, Kindt R, Legendre P, O'Hara B, Simpson GL, Solymos P, Stevens MHH, Wagner H. vegan: Community Ecology Package. R package version 1.15-0. In.; 2008.
21. Logares R, Audic S, Bass D, Bittner L, Boutte C, Christen R, Claverie JM, Decelle J, Dolan JR, Dunthorn M *et al*. Patterns of rare and abundant marine microbial eukaryotes. Current Biology. 2014; 24(8):813-821.
22. R-Development-Core-Team. R: A language and environment for statistical computing. Vienna, Austria: R Foundation for Statistical Computing; 2008.
23. Wickham H. ggplot2: Elegant Graphics for Data Analysis: Springer-Verlag; 2009.
24. Becker RA, Wilks AR, Brownrigg R, Minka TP, Deckmyn A. maps: Draw Geographical Maps. In.; 2017.
25. Gerritsen H. mapplots: Data Visualisation on Maps. In.; 2014.
26. Legendre P, De Caceres M. Beta diversity as the variance of community data: dissimilarity coefficients and partitioning. Ecol Lett. 2013; 16(8):951-963.
27. Dray S, Blanchet G, Borcard D, Clappe S, Guenard G, Jombart T, Larocque G, Legendre P, Madi N, Wagner HH. adespatial: Multivariate Multiscale Spatial Analysis. In.; 2017.
28. Schloss PD, Westcott SL, Ryabin T, Hall JR, Hartmann M, Hollister EB, Lesniewski RA, Oakley BB, Parks DH, Robinson CJ *et al*. Introducing mothur: open-source, platform-independent, community-supported software for describing and comparing microbial communities. Appl Environ Microbiol. 2009; 75(23):7537-7541.
29. Capella-Gutierrez S, Silla-Martinez JM, Gabaldon T. trimAl: a tool for automated alignment trimming in large-scale phylogenetic analyses. Bioinformatics. 2009; 25(15):1972-1973.

30. Price MN, Dehal PS, Arkin AP. FastTree: computing large minimum evolution trees with profiles instead of a distance matrix. Mol Biol Evol. 2009; 26(7):1641-1650.
31. Kembel SW, Cowan PD, Helmus MR, Cornwell WK, Morlon H, Ackerly DD, Blomberg SP, Webb CO. Picante: R tools for integrating phylogenies and ecology. Bioinformatics. 2010; 26(11):1463-1464.
32. Paradis E, Claude J, Strimmer K. APE: Analyses of Phylogenetics and Evolution in R language. Bioinformatics. 2004; 20(2):289-290.
33. Chen J, Bittinger K, Charlson ES, Hoffmann C, Lewis J, Wu GD, Collman RG, Bushman FD, Li H. Associating microbiome composition with environmental covariates using generalized UniFrac distances. Bioinformatics. 2012; 28(16):2106-2113.
34. Stegen JC, Lin X, Fredrickson JK, Chen X, Kennedy DW, Murray CJ, Rockhold ML, Konopka A. Quantifying community assembly processes and identifying features that impose them. ISME J. 2013; 7(11):2069-2079.
35. Stegen JC, Lin X, Konopka AE, Fredrickson JK. Stochastic and deterministic assembly processes in subsurface microbial communities. ISME J. 2012; 6(9):1653-1664.
36. Zhou J, Ning D. Stochastic Community Assembly: Does It Matter in Microbial Ecology? Microbiol Mol Biol Rev. 2017; 81(4):e00002-00017.
37. Chase JM, Kraft NJB, Smith KG, Vellend M, Inouye BD. Using null models to disentangle variation in community dissimilarity from variation in α-diversity. Ecosphere. 2011; 2(2):1-11.
38. Longhurst AR. Ecological Geography of the Sea: Academic Press; 2007.
39. Estrada M, Delgado M, Blasco D, Latasa M, Cabello AM, Benitez-Barrios V, Fraile-Nuez E, Mozetic P, Vidal M. Phytoplankton across Tropical and Subtropical Regions of the Atlantic, Indian and Pacific Oceans. PLoS One. 2016; 11(3):e0151699.
40. Catalá TS, Álvarez-Salgado XA, Otero J, Iuculano F, Companys B, Horstkotte B, Romera-Castillo C, Nieto-Cid M, Latasa M, Morán XAG *et al*. Drivers of fluorescent dissolved organic matter in the global epipelagic ocean. Limnology and Oceanography. 2016; 61(3):1101-1119.
41. Pérez-Mazuecos I. **Exopolymer particles in the ocean: production by microorganisms, carbon export and mesopelagic respiration**. *PhD Thesis.* University of Granada; 2015.
42. Catalá TS, Reche I, Ramón CL, López-Sanz À, Álvarez M, Calvo E, Álvarez-Salgado XA. Chromophoric signatures of microbial by-products in the dark ocean. Geophysical Research Letters. 2016; 43(14):7639-7648.
43. Fernandez-Castro B, Mourino-Carballido B, Maranon E, Choucino P, Gago J, Ramirez T, Vidal M, Bode A, Blasco D, Royer SJ *et al*. Importance of salt fingering for new nitrogen supply in the oligotrophic ocean. Nature communications. 2015; 6:8002.
44. Reshef DN, Reshef YA, Finucane HK, Grossman SR, McVean G, Turnbaugh PJ, Lander ES, Mitzenmacher M, Sabeti PC. Detecting novel associations in large data sets. Science. 2011; 334(6062):1518-1524.
