# Supplementary material for: Disentangling the mechanisms shaping the surface ocean microbiota
Source: Microbiome. 2020 Apr 20;8:55. doi: 10.1186/s40168-020-00827-8 (PMC7171866; doi:10.1186/s40168-020-00827-8)
Supplement: Supplementary file 10 — Additional file 9: Figure S6. Species association networks for the tropical and subtropical surface-ocean microbiota as inferred from the Malaspina dataset. Left-hand side: Association networks of picoeukaryotes and prokaryotes considering positive (red) and negative (blue) correlations in panels A) [Eukaryotic Network (+-e)] and B) [Prokaryotic Network (+-e)], and only positive correlations in C) [Eukaryotic Network (+e)] and D) [Prokaryotic Network (+e)]. On the right-hand side, we present an alternative visualization of the network as well as the following network characteristics: number of nodes (n), number of edges with positive correlation (+e) and negative correlation (-e), average degree (avg. d), average path length (avg. l), global transitivity (t), number of modules with at least 3 nodes (m) and the number of nodes in each of those modules (sizes: n). The smaller network visualization on the right-hand side groups the nodes according to the modules. The colors of nodes in Left- and Right-hand side networks indicate the modules to which they belong (NB: colors in panels A, B, C & D are independent of each other). [file 40168_2020_827_MOESM9_ESM.pdf]

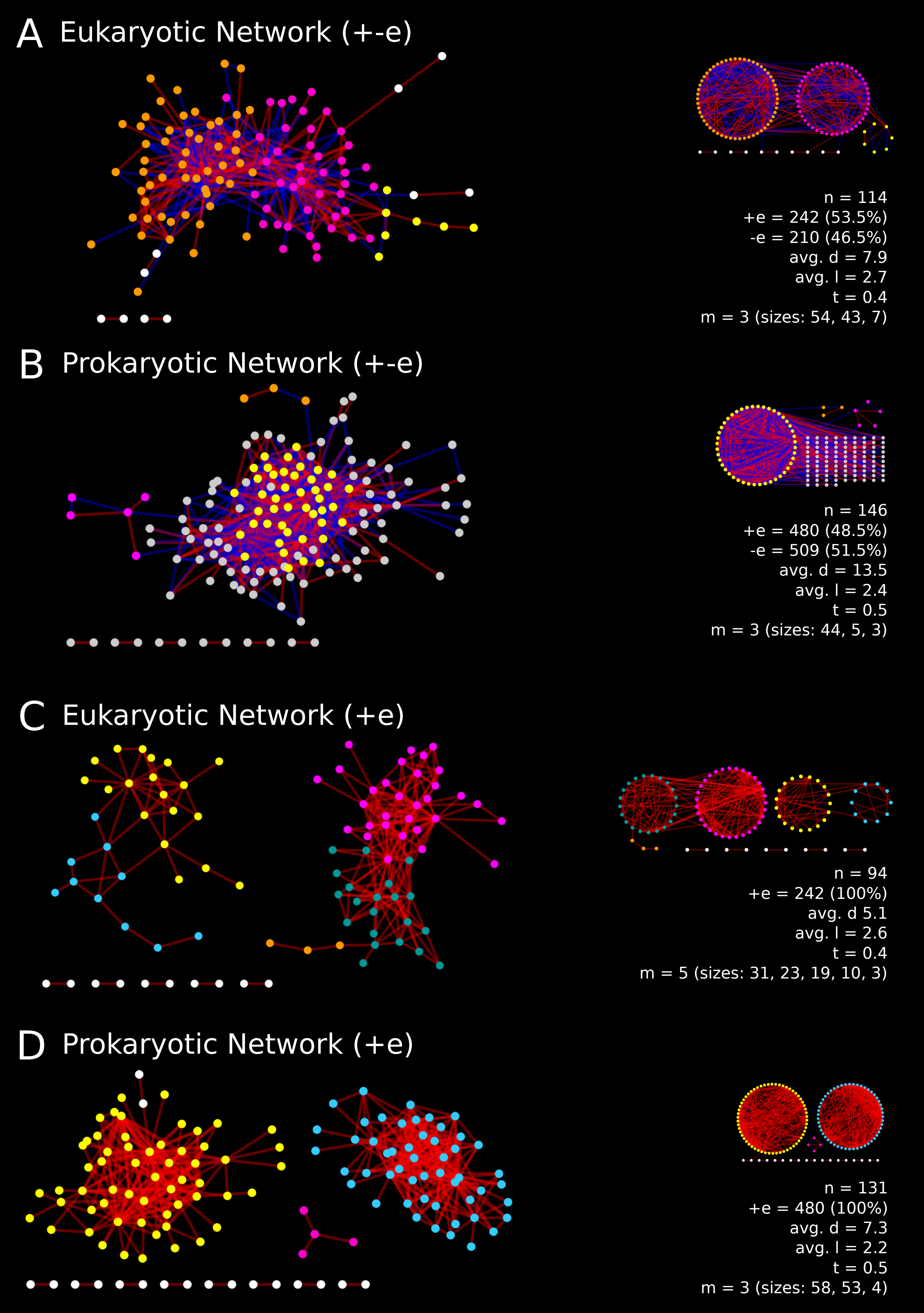
A Eukaryotic Network (+-e)
n = 114
+e = 242 (53.5%)
-e = 210 (46.5%)
avg. d = 7.9
avg. l = 2.7
t = 0.4
m = 3 (sizes: 54, 43, 7)
B Prokaryotic Network (+-e)
n = 146
+e = 480 (48.5%)
-e = 509 (51.5%)
avg. d = 13.5
avg. l = 2.4
t = 0.5
m = 3 (sizes: 44, 5, 3)
C Eukaryotic Network (+e)
n = 94
+e = 242 (100%)
avg. d 5.1
avg. l = 2.6
t = 0.4
m = 5 (sizes: 31, 23, 19, 10, 3)
D Prokaryotic Network (+e)
n = 131
+e = 480 (100%)
avg. d = 7.3
avg. l = 2.2
t = 0.5
m = 3 (sizes: 58, 53, 4)
