# Supplementary material for: Disentangling the mechanisms shaping the surface ocean microbiota
Source: Microbiome. 2020 Apr 20;8:55. doi: 10.1186/s40168-020-00827-8 (PMC7171866; doi:10.1186/s40168-020-00827-8)
Supplement: Supplementary file 11 — Additional file 10: Table S3. Summary of association networks from the Malaspina dataset based on SparCC. [file 40168_2020_827_MOESM10_ESM.docx]

**Table S3.** Summary of association networks from the *Malaspina* dataset based on SparCC [1].

|  | **Connected nodes**^1^ | **Positive edges**^2^ | | **Negative edges**^3^ | **Average degree**^4^ | **Transitivity (global)**^5^ | | **Average path length**^6^ | **Number of cliques (≥3 nodes)**^7^ | **Mean clique size**^8^ | **Number of modules**  **(≥3 nodes)**^9^ |
| --- | --- | --- | --- | --- | --- | --- | --- | --- | --- | --- | --- |
| **Eukaryotes (+-e)** | 114 (17.5%) | | 242 (53.5%) | 210 (46.5%) | 7.9 | | 0.4 | 2.7 | 120 | 5.4 | 3 (54,43,7) |
| **Prokaryotes (+-e)** | 146 (32.6%) | | 480 (48.5%) | 509 (51.5%) | 13.5 | 0.5 | | 2.4 | 262 | 11.4 | 3 (44,5,3) |
| **Eukaryotes (+e)** | 94 (14.4%) | | 242 (100%) | - | 5.1 | 0.4 | | 2.6 | 58 | 4.3 | 5 (31,23,19,10,3) |
| **Prokaryotes (+e)** | 131 (29.2%) | | 480 (100%) | - | 7.3 | 0.5 | | 2.2 | 103 | 6.2 | 3 (58,53,4) |

The eukaryotic and prokaryotic networks included 651 and 448 nodes (OTUs_-99%_) respectively, featuring absolute correlation scores >0.3 with p<0.01. Networks considering associations (edges) with both positive and negative correlation scores (+-e) as well as networks including positive correlation scores only (+e) are indicated. ^1^ Number of nodes with at least one edge as well as the percentage they represent of all the analysed nodes. ^2^ Number of edges with a positive correlation score and their percentage. ^3^ Number of edges with a negative correlation score and their percentage. ^4^ The degree indicates the number of edges connected to a node; the average degree refers to the sum of all degrees divided by the number of connected nodes. ^5^Transitivity measures the probability that two nodes that are connected to a third node are also connected (also known as Clustering Coefficient). ^6^The Average Path Length is the mean shortest distance between any pair of nodes in the network. ^7^ Cliques represent fully connected subnetworks of a network; the number of cliques with at least 3 nodes is indicated. ^8^ Average number of nodes in the network cliques of at least 3 nodes. ^9^ Number of modules (highly connected areas of a network) with at least 3 nodes calculated with the method considering edge betweenness [2]; the size of each module, in terms of number of nodes, is indicated.

**REFERENCES**

1. Friedman J, Alm EJ. Inferring correlation networks from genomic survey data. PLoS Comput Biol. 2012; 8(9):e1002687.

2. Girvan M, Newman ME. Community structure in social and biological networks. Proc Natl Acad Sci U S A. 2002; 99(12):7821-7826.
