# Supplementary material for: Disentangling the mechanisms shaping the surface ocean microbiota
Source: Microbiome. 2020 Apr 20;8:55. doi: 10.1186/s40168-020-00827-8 (PMC7171866; doi:10.1186/s40168-020-00827-8)
Supplement: Supplementary file 12 — Additional file 11: Table S4. Summary of significant OTUs-99% associations using MIC for the Malaspina dataset. [file 40168_2020_827_MOESM11_ESM.docx]

**Table S4.** Summary of significant OTUs_-99%_ associations using MIC [1] for the *Malaspina* dataset.

|  | **Associations**^1^ | **% OTUs (#)**^2^ | **Abundance (%)**^3^ | **MIC-⍴^2^ > 0.2 (%**)^4^ |
| --- | --- | --- | --- | --- |
| **Eukaryote – Eukaryote (MIC>0.4)^5^** | 121 | 15 (97) | 16 | 51 |
| **Eukaryote – Eukaryote (MIC>0.3)^5^** | 914 | 60 (388) | 61 | 71 |
| **Prokaryote – Prokaryote (MIC>0.4)^5^** | 618 | 51 (229) | 71 | 54 |
| **Prokaryote – Prokaryote (MIC>0.3)^5^** | 3,163 | 87 (389) | 83 | 53 |
| **Eukaryote – Prokaryote (MIC>0.4)^5^** | 143 | - | - | 75 |
| **Eukaryote – Prokaryote (MIC>0.3)^5^** | 1,507 | - | - | 73 |
| **Eukaryotes in Eukaryote – Prokaryote associations (MIC>0.4) ^5^** | - | 7 (49) | 8 | - |
| **Eukaryotes in Eukaryote – Prokaryote associations (MIC>0.3) ^5^** | - | 47 (302) | 51 | - |
| **Prokaryotes in Eukaryote – Prokaryote associations (MIC>0.4) ^5^** | - | 12 (52) | 46 | - |
| **Prokaryotes in Eukaryote – Prokaryote associations (MIC>0.3) ^5^** | - | 54 (244) | 73 | - |
| **Eukaryotes – Environment (MIC>0.4)^6^** | 269 | 1 (159) | 15 | 87 |
| **Eukaryotes – Environment (MIC>0.3)^6^** | 3,165 | 8 (1430) | 74 | 92 |
| **Prokaryotes – Environment (MIC>0.4)^6^** | 403 | 3 (212) | 30 | 78 |
| **Prokaryotes – Environment (MIC>0.3)^6^** | 3,186 | 17 (1192) | 88 | 85 |

All MIC values have a p<0.05. ^1^ Number of associations (NB: OTUs may feature more than one association). ^2^ Percentage of OTUs involved in associations; corresponding OTU numbers are given within parentheses. ^3^ Percentage of total abundance of OTUs involved in associations. ^4^ Percentage of non-linear associations (MIC-⍴^2^ >0.2) out of all associations^1^. ^5^ Analyses included OTUs with >100 reads from sub-sampled OTU tables (Picoeukaryotes: 648 OTUs; Prokaryotes:448 OTUs).  ^6^ Analyses were done with an OTU table including all OTUs. Prokaryotes: Total OTUs = 7,025, Total reads: 489,039; Eukaryotes: Total OTUs = 18,775, Total reads: 491,260. Included environmental parameters are indicated in *Environmental datasets* above.

**REFERENCES**

1. Reshef DN, Reshef YA, Finucane HK, Grossman SR, McVean G, Turnbaugh PJ, Lander ES, Mitzenmacher M, Sabeti PC. Detecting novel associations in large data sets. Science. 2011; 334(6062):1518-1524.
