# Supplementary material for: Disentangling the mechanisms shaping the surface ocean microbiota
Source: Microbiome. 2020 Apr 20;8:55. doi: 10.1186/s40168-020-00827-8 (PMC7171866; doi:10.1186/s40168-020-00827-8)
Supplement: Supplementary file 13 — Additional file 12: Table S5. Summary of significant OTUs-99% associations for the TARA Oceans dataset based on MIC. [file 40168_2020_827_MOESM12_ESM.docx]

**Table S5.** Summary of significant OTUs_-99%_ associations with MIC > 0.5 for the *TARA Oceans* dataset based on MIC.

|  | **Associations^1^** | **% OTUs (#)^2^** | **Abundance (%)^3^** | **MIC-⍴^2^ > 0.2 (%)^4^** |
| --- | --- | --- | --- | --- |
| **Eukaryote – Eukaryote (MIC>0.8)** | 747 | 21.5 (437) | 22.7 | 91.3 |
| **Eukaryote – Eukaryote (MIC>0.7)** | 3,238 | 50.7 (1,028) | 66.4 | 89.6 |
| **Eukaryote – Eukaryote (MIC>0.5)** | 49,839 | 96.5 (1,956) | 99.6 | 88.1 |
| **Prokaryote – Prokaryote (MIC>0.8)** | 2,270 | 46.6 (771) | 69.0 | 23.6 |
| **Prokaryote – Prokaryote (MIC>0.7)** | 6,698 | 72.6 (1,203) | 84.9 | 37.2 |
| **Prokaryote – Prokaryote (MIC>0.5)** | 73,705 | 97.2 (1,611) | 99.2 | 63.4 |
| **Eukaryote – Environment (MIC>0.7)** | 248 | 1.96 (199) | 4.0 | 99.1 |
| **Eukaryote – Environment (MIC>0.5)** | 2,811 | 19.3 (1957) | 28.5 | 94.8 |
| **Prokaryotes – Environment (MIC>0.7)** | 66 | 1.0 (50) | 0.99 | 92.4 |
| **Prokaryotes – Environment (MIC>0.5)** | 1,099 | 14.7 (740) | 17.9 | 72.6 |

^1^ Number of associations (NB: OTUs may feature more than one association). ^2^ Percentage of OTUs involved in associations; corresponding OTU numbers are given within parentheses. ^3^ Percentage of total abundance of OTUs involved in associations.  ^4^ Percentage of non-linear associations (MIC-⍴^2^ >0.2) out of all associations^1^
