# Supplementary material for: Disentangling the mechanisms shaping the surface ocean microbiota
Source: Microbiome. 2020 Apr 20;8:55. doi: 10.1186/s40168-020-00827-8 (PMC7171866; doi:10.1186/s40168-020-00827-8)
Supplement: Supplementary file 15 — Additional file 14: Table S6. Significant MIC associations (MIC > 0.5) between OTUs and environmental parameters in the TARA Oceans dataset. [file 40168_2020_827_MOESM14_ESM.docx]

**Table S6.** Significant MIC associations (MIC > 0.5) between OTUs and environmental parameters in the *TARA Oceans* dataset.

| **Environmental variable** | **Number of OTUs^1^** | **%^2^** | **OTU abundance (# reads)^3^** | **%^4^** |
| --- | --- | --- | --- | --- |
|  |  |  |  |  |
| **PROKARYOTES** |  |  |  |  |
| Temperature (ºC) | 441 | 40.1 | 1,111,758 | 37.4 |
| Salinity | 4 | 0.4 | 6,365 | 0.2 |
| Oxygen (μmol/kg) | 555 | 50.5 | 1,473,130 | 49.5 |
| NO_3_ (μmol/L) | 2 | 0.2 | 7,189 | 0.2 |
| NO_2_ (μmol/L) | 37 | 3.4 | 176,421 | 5.9 |
| PO_4_ (μmol/L) | 25 | 2.3 | 66,969 | 2.2 |
| NO_2_NO_3_ (μmol/L) | 31 | 2.8 | 125,608 | 4.2 |
| SI (μmol/L) | 4 | 0.4 | 7,209 | 0.2 |
|  |  |  |  |  |
| **MICROBIAL EUKARYOTES** |  |  |  |  |
| Temperature (ºC) | 836 | 29.7 | 7,166,962 | 27.7 |
| Salinity | 464 | 16.5 | 4,172,647 | 16.1 |
| Oxygen (μmol/kg) | 662 | 23.5 | 4,024,073 | 15.5 |
| NO_3_ (μmol/L) | 471 | 16.7 | 4,050,804 | 15.6 |
| PAR | 106 | 3.8 | 717,541 | 2.8 |
| Chlorophyll a (mg/m^3^) | 272 | 9.7 | 5,747,883 | 22.2 |

^1^ Number of OTUs associated to each environmental parameter. ^2^ Percentage of total OTUs. ^3^ Number of reads represented by the OTUs. ^4^ Percentage of total reads. NB: percentage columns do not sum 100% as OTUs can be involved in associations with more than one environmental variable or they can present no associations with them.
