# Supplementary material for: Disentangling the mechanisms shaping the surface ocean microbiota
Source: Microbiome. 2020 Apr 20;8:55. doi: 10.1186/s40168-020-00827-8 (PMC7171866; doi:10.1186/s40168-020-00827-8)
Supplement: Supplementary file 18 — Additional file 17: Table S7. The 36 Malaspina stations (out of 120) featuring significant (p < 0.05) Local Contributions to Beta Diversity (LCBD) in prokaryotes and/or picoeukaryotes. [file 40168_2020_827_MOESM17_ESM.docx]

**Table S7.** The 36 *Malaspina* stations (out of 120) featuring significant (p<0.05) Local Contributions to Beta Diversity (LCBD [1]) in prokaryotes and/or picoeukaryotes.

| **#** | **Station ID** | **Sample** | **Prokaryotes** | **Picoeukaryotes** |
| --- | --- | --- | --- | --- |
| 1 | 1 | ST_1_MD28 | * | N.S. |
| 2 | 7 | ST_7_MD98 | N.S. | * |
| 3 | **27** ^a^ | ST_27_MD458 | * | * |
| 4 | **29** ^a^ | ST_29_MD506 | * | * |
| 5 | 30 | ST_30_MD528 | N.S. | * |
| 6 | **37** ^a^ | ST_37_MD646 | * | * |
| 7 | **38** ^a^ | ST_38_MD664 | * | * |
| 8 | 39 | ST_39_MD684 | * | N.S. |
| 9 | **40** | ST_40_MD712 | * | * |
| 10 | **41** | ST_41_MD734 | * | * |
| 11 | **43** ^a^ | ST_43_MD753 | * | * |
| 12 | **44** | ST_44_MD778 | * | * |
| 13 | 45 ^a^ | ST_45_MD806 | N.S. | * |
| 14 | 53 | ST_53_MD962 | N.S. | * |
| 15 | **54** | ST_54_MD985 | * | * |
| 16 | 58 | ST_58_MD1080 | N.S. | * |
| 17 | 67 | ST_67_MD1246 | N.S. | * |
| 18 ^b^ | **71** | ST_71_MD1318 ^b^ | * | * |
| 18 ^b^ | 71 | ST_71_MD1324 ^b^ | * | N.S. |
| 19 | 72 | ST_72_MD1331 | * | N.S. |
| 20 | 73 | ST_73_MD1354 | * | N.S. |
| 21 | **74** | ST_74_MD1368 | * | * |
| 22 | 75 | ST_75_MD1398 | * | N.S. |
| 23 | 76 | ST_76_MD1421 | * | N.S. |
| 24 | 77 | ST_77_MD1425 | N.S. | * |
| 25 | 89 | ST_89_MD1629 | * | N.S. |
| 26 | 92 | ST_92_MD1672 | * | N.S. |
| 27 | 94 | ST_94_MD1724 | * | N.S. |
| 28 | **95** | ST_95_MD1744 | * | * |
| 29 | 96 | ST_96_MD1772 | * | N.S. |
| 30 | 97 | ST_97_MD1798 | N.S. | * |
| 31 | **124** | ST_124_MD2332 | * | * |
| 32 | **125** | ST_125_MD2340 | * | * |
| 33 | 130 | ST_130_MD2474 | N.S. | * |
| 34 | 132 | ST_132_MD2562 | N.S. | * |
| 35 | **133** ^a^ | ST_133_MD2594 | * | * |
| 36 | **135** ^a^ | ST_135_MD2662 | * | * |

* LCBD p<0.05. N.S.: Non-significant. Both picoeukaryotes and prokaryotes featured 26 stations each with LCBD p<0.05, totaling 36 stations. A total of 16 stations displayed samples with LCBD p<0.05 for both prokaryotes and picoeukaryotes (shown in **bold**). ^a^ Stations identified also in sequential β diversity analyses (8 stations) as points of abrupt β diversity change. ^b^ Two samples from the same station. OTUs_-99%_ were used in this analysis.

**REFERENCES**

1. Legendre P, De Caceres M. Beta diversity as the variance of community data: dissimilarity coefficients and partitioning. Ecol Lett. 2013; 16(8):951-963.
