# Supplementary material for: Disentangling the mechanisms shaping the surface ocean microbiota
Source: Microbiome. 2020 Apr 20;8:55. doi: 10.1186/s40168-020-00827-8 (PMC7171866; doi:10.1186/s40168-020-00827-8)
Supplement: Supplementary file 19 — Additional file 18: Table S8.Malaspina eukaryotic and prokaryotic reads and OTUs-99% analysed during different steps of our in-house workflow. [file 40168_2020_827_MOESM18_ESM.docx]

**Table S8.** *Malaspina* eukaryotic and prokaryotic reads and OTUs_-99%_ analysed during different steps of our in-house workflow [1].

| **Processing step** | **Picoeukaryotes** | **Prokaryotes** |
| --- | --- | --- |
| Total reads | 35,695,530 (100%) | 8,564,836 (100%) |
| Merged reads (PEAR) | 34,768,276 (97.4%) | 8,516,553 (99.4%) |
| Reads passing quality control (max_ee =0.5) | 19,230,661 (55.3%) | 7,234,052 (84.9%) |
| **Reads after HMM^1^ (rDNA validation)^2^** | **19,230,187 (99.9%)** | **7,234,049 (99.9%)** |
| De-replicated reads (incl. singletons) | 5,644,422 | 1,748,584 |
| Singletons | 4,526,205 | 1,450,578 |
| De-replicated reads (without singletons) | 1,118,217 | 298,006 |
| Chimeric reads (de-novo detection during Uparse) | 192,186 (17.2%) | 105,470 (35.4%) |
|  |  |  |
| Total OTUs (99% clustering UPARSE) | 51,571 (100%) | 13,300 (100%) |
| Chimeric OTUs (reference-based: positive) | 6,605 (12.8%) | 2,413 (18.1%) |
| Chimeric OTUs (reference-based: uncertain) | 2,461 (4.8%) | 729 (5.5%) |
| **OTUs 99% - Non-Chimeric** | **42,505 (82.4%)** | **10,158 (76.4%)** |
|  |  |  |
| **Reads^2^ mapped back to OTUs (99% similarity)** | **16,460,248 (85.6%)** | **5,697,779 (78.8%)** |
| Reads^2^ not mapping back to OTUs (99% similarity) | 2,769,939 (14.4%) | 1,536,270 (21.2%) |
|  |  |  |

^1^ Hidden Markov Models.  ^2^Indicate the reads that were mapped back to OTUs.

**REFERENCES**

1. Logares R. Workflow for Analysing MiSeq Amplicons based on Uparse v1.5. In*.*: <https://doi.org/10.5281/zenodo.259579>; 2017.
