# Supplementary material for: Disentangling the mechanisms shaping the surface ocean microbiota
Source: Microbiome. 2020 Apr 20;8:55. doi: 10.1186/s40168-020-00827-8 (PMC7171866; doi:10.1186/s40168-020-00827-8)
Supplement: Supplementary file 20 — Additional file 19: Figure S10. Phylogenetic signal was detected across short phylogenetic distances for both the 16S and 18S rRNA-gene markers as indicated by phylogenetic mantel correlograms (Malaspina dataset). Phylogenetic signal was tested using temperature and fluorescence, the two variables that explain the highest fraction of community variance. Solid and open squares indicate significant and nonsignificant (using p=0.05) correlations respectively between environmental similarity (in terms of temperature and fluorescence) and phylogenetic relatedness. Correlations that are significantly positive indicate that the phylogenetic distance between OTUs-99% increases as environmental similarity decreases for the phylogenetic range being analysed. Phylogenetic distances were measured as abundance-weighted β-Mean Nearest Taxon Distances (βMNTD). [file 40168_2020_827_MOESM19_ESM.pdf]

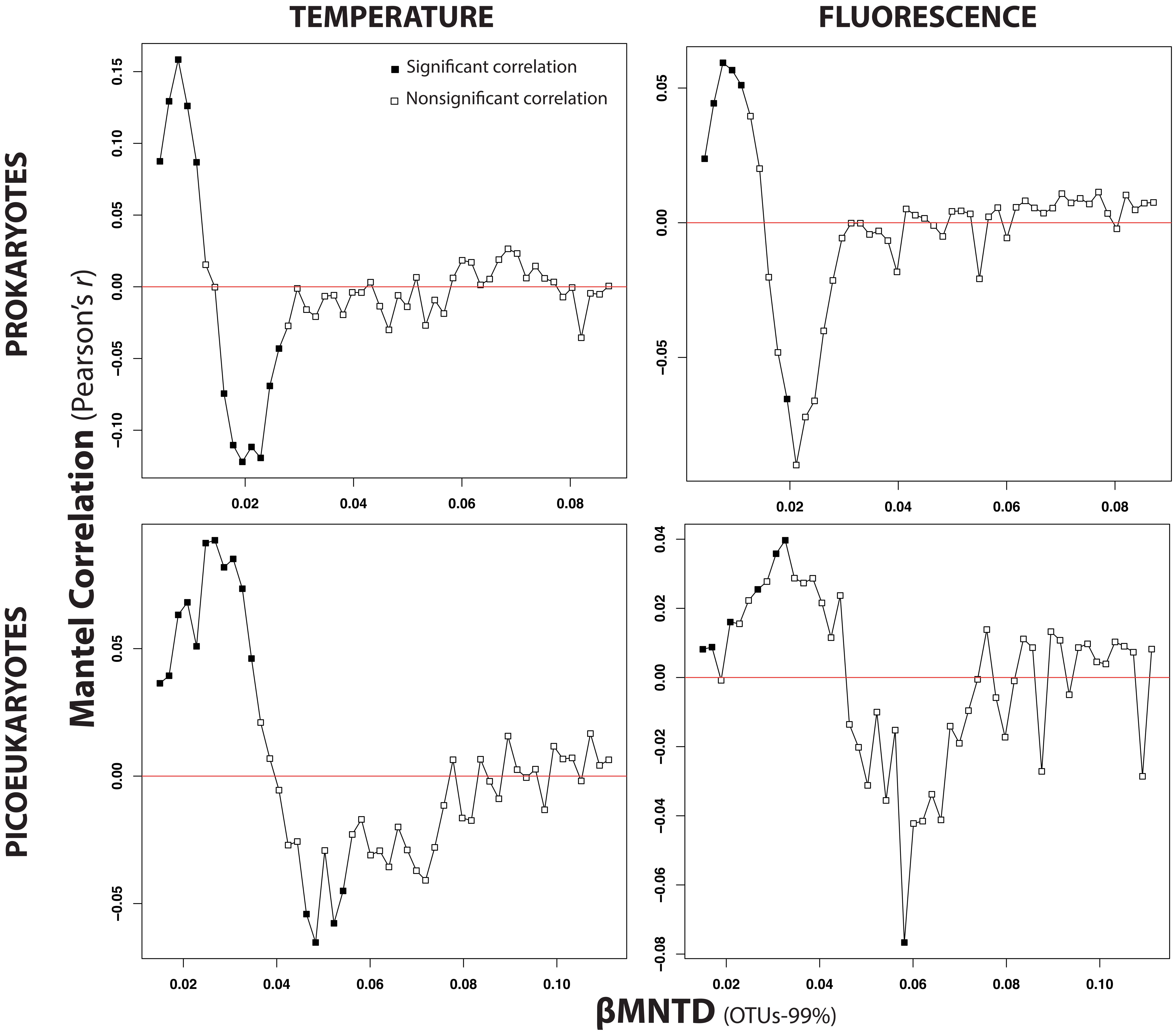

TEMPERATURE
FLUORESCENCE
PROKARYOTES
PICOEUKARYOTES
Mantel Correlation (Pearson's r)
βMNTD (OTUs-99%)
Significant correlation
Nonsignificant correlation
