# Supplementary material for: Disentangling the mechanisms shaping the surface ocean microbiota
Source: Microbiome. 2020 Apr 20;8:55. doi: 10.1186/s40168-020-00827-8 (PMC7171866; doi:10.1186/s40168-020-00827-8)
Supplement: Supplementary file 21 — Additional file 20: Figure S11. Same as Figure S10, Additional file 19 but using OTUs-ASVs. Solid and open squares indicate significant and nonsignificant (using p=0.05) correlations respectively between environmental similarity (in terms of temperature and fluorescence) and phylogenetic relatedness. Correlations that are significantly positive indicate that the phylogenetic distance between OTUs-ASVs increases as environmental similarity decreases for the phylogenetic range being analysed. Phylogenetic distances were measured as abundance-weighted β-Mean Nearest Taxon Distances (βMNTD). [file 40168_2020_827_MOESM20_ESM.pdf]

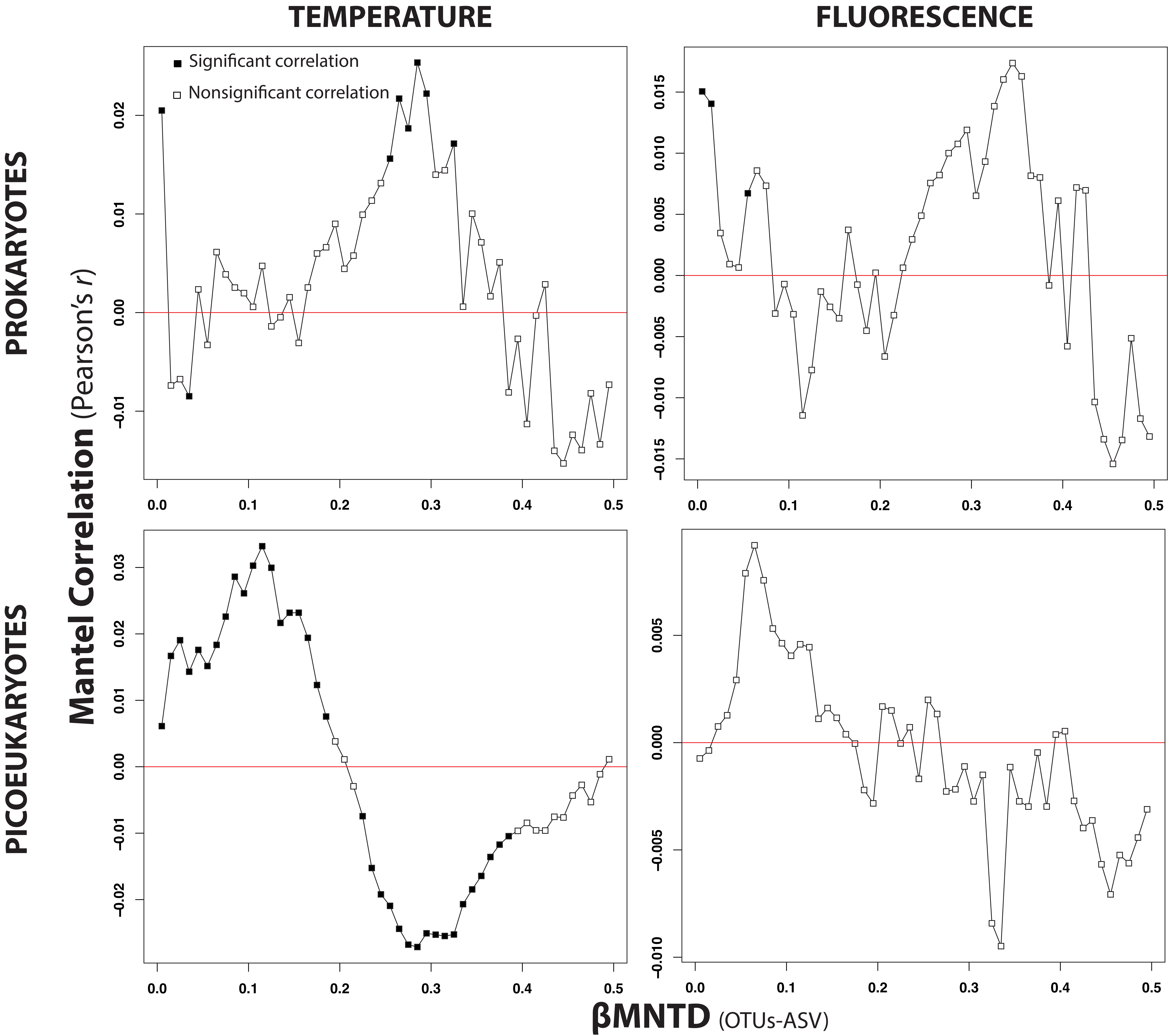

TEMPERATURE
FLUORESCENCE
PROKARYOTES
PICOEUKARYOTES
Mantel Correlation (Pearson's r)
Significant correlation
Nonsignificant correlation
βMNTD (OTUs-ASV)
